# Na-doped ruthenium perovskite electrocatalysts with improved oxygen evolution activity and durability in acidic media

**DOI:** 10.1038/s41467-019-09791-w

**Published:** 2019-05-03

**Authors:** María Retuerto, Laura Pascual, Federico Calle-Vallejo, Pilar Ferrer, Diego Gianolio, Amaru González Pereira, Álvaro García, Jorge Torrero, María Teresa Fernández-Díaz, Peter Bencok, Miguel A. Peña, José Luis G. Fierro, Sergio Rojas

**Affiliations:** 1 Grupo de Energía y Química Sostenible, Instituto de Catálisis y Petroleoquímica, CSIC. C/ Marie Curie 2, 28049 Madrid, Spain; 2 Instituto de Catálisis y Petroleoquímica, CSIC. C/ Marie Curie 2, 28049 Madrid, Spain; 3 Departament de Ciència de Materials i Química Física & Institut de Química Teòrica i Computacional (IQTCUB), Universitat de Barcelona, 08028 Barcelona, Spain; 4 Diamond Light Source, Harwell Science and Innovation Campus, Chilton, Didcot OX11 0DE UK; 5 Institut Laue-Langevin, BP156X, F-38042 Grenoble, France

**Keywords:** Solid-state chemistry, Renewable energy, Electrocatalysis

## Abstract

The design of active and durable catalysts for the H_2_O/O_2_ interconversion is one of the major challenges of electrocatalysis for renewable energy. The oxygen evolution reaction (OER) is catalyzed by SrRuO_3_ with low potentials (ca. 1.35 V_RHE_), but the catalyst’s durability is insufficient. Here we show that Na doping enhances both activity and durability in acid media. DFT reveals that whereas SrRuO_3_ binds reaction intermediates too strongly, Na doping of ~0.125 leads to nearly optimal OER activity. Na doping increases the oxidation state of Ru, thereby displacing positively O p-band and Ru d-band centers, weakening Ru-adsorbate bonds. The enhanced durability of Na-doped perovskites is concomitant with the stabilization of Ru centers with slightly higher oxidation states, higher dissolution potentials, lower surface energy and less distorted RuO_6_ octahedra. These results illustrate how high OER activity and durability can be simultaneously engineered by chemical doping of perovskites.

## Abstract (web summary)

While water splitting may afford a renewable means to store energy in chemical bonds, water oxidation catalysts suffer from poor stabilities in acidic media. Here, authors show sodium doping of strontium ruthenate to improve the catalytic durability while maintaining a high O_2_ evolution activity.

## Introduction

Electrochemical technologies promise the efficient and versatile storage and use of renewable energy in cycles that do not generate harmful by-products^1^. Therefore, they are expected to become major players in the shift towards circular economy and energy systems, once their present problems are solved^2^. For instance, the oxygen evolution reaction (OER) in which H_2_O is oxidized to O_2_ is crucial in electrolyzers and in the charging of metal-air batteries. To compensate the sluggish kinetics of the OER, high overpotentials are needed in practical applications, causing severe energy losses^3^. Ir-based materials are the state-of-the-art OER catalysts^4,5^, which owing to their high price and scarcity must be replaced to facilitate wider implementation of electrolyzers. Functional oxides such as perovskites are promising OER electrocatalysts, especially in alkaline media^6–10^. Recently, high OER activities in alkaline electrolyte have been reported for thin films of SrRuO_3_ (0.1 mA cm^−2^ at 1.33 V vs. RHE)^11^. However, this material loses its activity after only two cycles. The lack of stability of Ru-based oxides appears to be related to the transformation of Ru^4+^ into Ru^>4+^ at high voltages (above 1.3–1.4 V)^12,13^ leading to the decomposition of the perovskite, which starts with Sr dissolution followed by the collapse of the mixed-oxide structure^11^. Very few examples of perovskites with high OER activity in acid exist. For instance, SrRuO_3_ nanopowders^13^ (initial 30 Ag^−1^ at 1.37 V), bulk iridium-based double perovskites^14^ (10 mAcm^−2^_oxide_ at 1.6 V), SrIrO_3_/IrO_*x*_ thin films^15^ (10 mA cm^−2^_oxide_ at 1.5 V). On the other hand, Ir, Ru, IrO_2_ and RuO_2_ nanoparticles show high OER activity, Ru-based compounds present 2 mA cm^−2^ at 1.42 V and IrO_*x*_ ~ 10 mA cm^−2^_oxide_ at 1.52 V^16,17^. A grand challenge in electrocatalysis is, therefore, the design of perovskites with high and long-lasting OER activities in acid media.

Here we show that the OER activity and durability in acid of bulk SrRuO_3_ can be enhanced by Na^+^ doping in the Sr^2+^ position. Thus, Sr_0.95_Na_0.05_RuO_3_ and S_0.90_Na_0.10_RuO_3_ exhibit very high specific OER activity, with a potential of ~1.35 V (an overpotential of only 120 mV) at 0.5 mA cm^−2^_geo_. Thorough physical and chemical studies of fresh and used perovskites reveal that substituting Sr by Na increases the stability of the perovskite structure, thus preventing deactivation during repeated cycling. This result is also supported by DFT, demonstrating lower surface energy and higher dissolution potentials for Na-doped perovskites, which slows down the collapse of the perovskite structure.

## Results and discussion

### Composition, structure, and oxidation state

SrRuO_3_, Sr_0.95_Na_0.05_RuO_3_, and Sr_0.90_Na_0.10_RuO_3_ were prepared by sol–gel chemistry followed by thermal treatment in air, with an increase of the synthesis temperature as the content of Na increases (Methods section). The chemical composition of the samples was analyzed by PND, EDX, and ICP-OES (see Supplementary Note 1). Sr and Na contents of Sr0.936(7)/Na0.064(7) for *x* = 0.05 and Sr0.906(1)/Na0.094(1) for *x* = 0.10 were obtained from PND. As discussed below, STEM-EDX mappings confirm that Na is homogeneously distributed into the perovskites. Attempts to introduce higher Na, e.g., Sr_0.85_Na_0.15_RuO_3_ and Sr_0.80_Na_0.20_RuO_3_ were not successful. Thus, the actual content of Na in Sr_0.85_Na_0.15_RuO_3_ is *x* ~ 0.08, lower than the nominal value. This is probably because the higher synthesis temperature results in a partial evaporation of Na. In fact, Sr_0.80_Na_0.20_RuO_3_ could not be prepared. For what is worth, characterization details for Sr_0.85_Na_0.15_RuO_3_ are presented in the Supplementary Note 2, Supplementary Fig. 1, and Supplementary Tables 1, 2 and 3.

SrRuO_3_, Sr_0.95_Na_0.05_RuO_3_, and Sr_0.90_Na_0.10_RuO_3_ display perovskite structure (see x-ray diffraction (XRD) patterns, Supplementary Fig. 2). The cell volume decreases upon Na incorporation (Inset of Supplementary Fig. 2), justified by the smaller radii of Na^+^ and Ru^>4+^ compared to Sr^2+^ and Ru^4+^, respectively^18^, and in good agreement with previous literature^19^ and our DFT calculations (see the Computational Methods section).

**Fig. 1:**
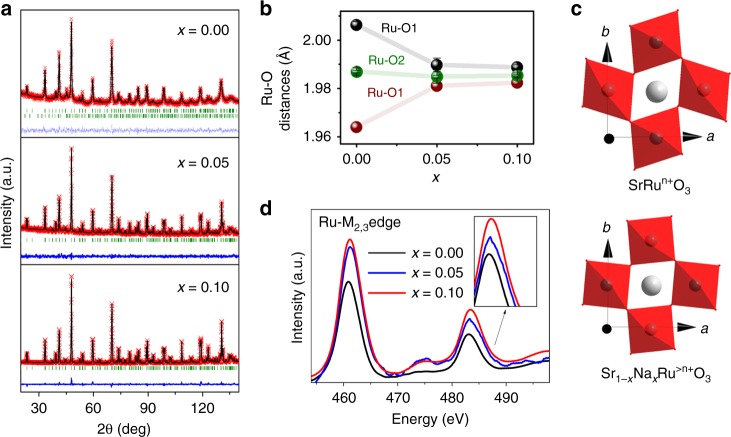
Crystallographic study of SrRuO_3_, Sr_0.95_Na_0.05_RuO_3_ and Sr_0.90_Na_0.10_RuO_3_. **a** Structural Rietveld refinements from PND data at room temperature. Observed (crosses), calculated (full line) and difference (bottom) profiles. Bragg lines correspond to the crystallographic structures. The second Bragg line on SrRuO_3_ corresponds to ~6% of Sr_4_Ru_2_O_3_. **b** Ruthenium-oxygen distances in the RuO_6_ octahedra. **c** Octahedral distortion in SrRuO_3_ (exaggerated to facilitate its visualization) compared to Na-doped samples. **d** Ru M_2,3_-edge XANES spectra for SrRuO_3_ (black), Sr_0.95_Na_0.05_RuO_3_ (blue) and Sr_0.90_Na_0.10_RuO_3_ (red). The inset shows the ~461 eV region with SrRuO_3_ spectrum displaced upward for clarity

Powder neutron diffraction (PND) is the most suitable technique to assess oxygen vacancies, atomic occupancies, structural distortions, atomic positions, bond distances, and angles of oxides. SrRuO_3_ and Na-doped samples adopt an orthorhombic perovskite structure with space group *Pbnm*, with Sr^2+^/Na^+^ located randomly at A sites and Ru^n+^ at B sites, as previously reported^19^. Fig. 1a shows good agreement between calculated and experimental PND data. The structural results obtained from PND Rietveld refinements are shown in Supplementary Table 4. First, we verified the lack of vacancies in A-sites and then we refined the Na occupancy into Sr-sites. The refined Na contents are close to the nominal values, in agreement with the values obtained by ICP-OES and the cell volume observed with XRD (Supplementary Note 1, Supplementary Fig. 2, and Supplementary Table 5). Figure 1b and Supplementary Fig. 3 show the Ru–O distances on the RuO_6_ octahedra. RuO_6_ octahedra in SrRuO_3_ are more distorted than in Sr_0.95_Na_0.05_RuO_3_ and Sr_0.90_Na_0.10_RuO_3_, with large *ab* in-plane deformation, shortened Ru-O1 distances (1.950(2) Å) and elongated Ru-O1 distances (2.006(2) Å). Note that this deformation has been previously reported for SrRuO_3_^20,21^. Conversely, Na-doped samples show very similar Ru–O distances (of ca. 1.98 Å) on the RuO_6_ octahedra, indicating that the octahedra are regular and not distorted as in SrRuO_3_ (Fig. 1c). The different octahedra distortion of SrRuO_3_ and Na-doped samples could be a consequence of the slight change of Ru oxidation states in Na-doped perovskites, as confirmed from XAS (see below). SrRuO_3_ contains Ru^4+^ cations (4*d*^4^) with relatively extended *d* orbitals. Ru^4+^ adopts a low spin configuration (t_2g_^3↑^t_2g_^1↓^e_g_^0^)^20^, favouring Jahn–Teller distortions that split the threefold t_2g_ into one lower *d*_*xz*_ orbital and two higher *d*_*yz*_ and *d*_*xy*_ orbitals. Such Jahn–Teller distortion causes the aforementioned elongated and shortened Ru–O distances. However, upon Na doping, a slight over-oxidation to Ru^>4+^ occurs resulting in the partial elimination of the fourth spin of the t_2g_, which could prevent the Jahn–Teller effect (or produce a switch to a high spin configuration) resulting in more regular octahedra. In addition, the higher temperatures used for the synthesis of the Na-doped perovskites could affect the different distortions. The implications of the actual structure of the perovskite for the OER performance are discussed below.

PND results also show that, within the error, the perovskites lack cationic and oxygen vacancies (Supplementary Table 4). Further experimental evidences of the absence of short- or long-range ordered oxygen vacancies were attained from HRTEM and selected area electron diffraction (SAED) patterns (Supplementary Fig. 4) in which diffraction spots for stoichiometric perovskites are the only ones observed. Moreover, DFT reveals large energies of formation of oxygen vacancies (bulk and surface), confirming the unlikely formation of such vacancies (Supplementary Note 3, Supplementary Fig. 5, and Supplementary Table 6). The lack of oxygen vacancies implies that Na incorporation is compensated by partial oxidation of Ru atoms.

The effect of Na doping in the structure and oxidation state of Ru was analyzed by x-ray absorption spectroscopy (XAS). We performed XAS in different energy ranges, soft and hard x-rays, to study the K-edge of Na, and the K-edges and M_2,3_-edges of Ru. The spectra of the Na K-edge confirm the presence of Na in the Na-doped perovskites (Supplementary Fig. 6). The strong overlap with the Sr L-edge background signal prevents an accurate estimation of the actual Na-content in each perovskite by this technique. In addition, the analysis of the Ru K-edge further confirms the incorporation of Na into the perovskites (Supplementary Note 4, and Supplementary Figs. 6b and 7). The Fourier transforms from the Ru K-edge EXAFS signals (phase non-corrected) shown in Supplementary Fig. 6b reveal that the signals for SrRuO_3_ are less intense than the ones of the Na-doped samples, indicating a higher distortion of the RuO_6_ octahedra in SrRuO_3_, in agreement with PND data.

The oxidation state of Ru was determined by the analysis of the Ru M_2,3_-edge. This edge is associated with the promotion of 3p to 4d orbitals in Ru and is more sensitive to changes in the d-states than the K-edge. As observed in Fig. 1d, the XANES signals of Sr_0.95_Na_0.05_RuO_3_ and Sr_0.90_Na_0.10_RuO_3_ appear at higher energies than SrRuO_3_. In addition, a slight broadening to the low energy region of the peaks of the Na-doped is observed. Both features are indicative of a higher oxidation state of Ru atoms in the Na-containing perovskites^22^.

### Electrochemical performance

The OER activity of SrRuO_3_, Sr_0.95_Na_0.05_RuO_3_, and Sr_0.90_Na_0.10_RuO_3_ was evaluated in O_2_-saturated 0.1 M HClO_4_ at 1600 rpm. The catalysts were deposited onto a glassy carbon electrode using an ink (Methods section). High surface area carbon was mixed with the perovskites (1:5 in weight) to improve the conductivity^23^.

**Fig. 2:**
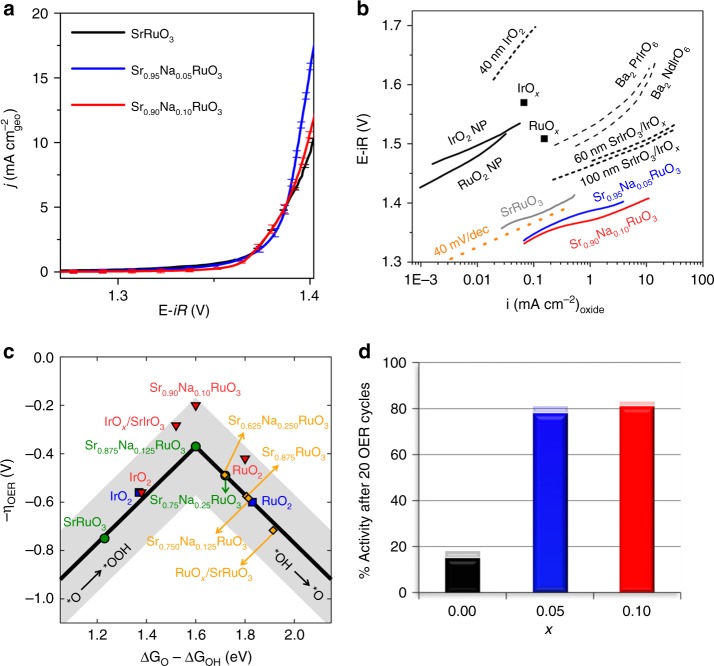
OER activity and durability. **a** Current densities (*j*) curves (SrRuO_3_ (black), Sr_0.95_Na_0.05_RuO_3_ (blue) and Sr_0.90_Na_0.10_RuO_3_ (red)) including the relative standard deviation (RSD). **b** TAFEL plots compared to the intrinsic activities of selected OER catalysts in acidic media. The data are taken from ref. ^14,15^. We represent the normalization using A_S_. A line corresponding to a Tafel slope of 40 mA/decade (magenta dotted line) serves as a guide to the eye. **c**, OER Volcano-type activity plot. Data in green for Sr_1-*x*_Na_*x*_RuO_3_, orange (partially dissolved perovskites, see also Supplementary Fig. 14c) and RuO_2_ (blue) are from this study, data for IrO_2_ (blue) is from ref. ^60^. Data in red come from experiments (this work; refs. ^15,30^), following the approach of Jaramillo and co-workers^36^, which correlates DFT-calculated descriptors with experimental overpotentials at 1 mA/cm^2^_oxide_. Error bands of ± 0.2 V surround the lines to account for the accuracy of the calculations. **d** Percentage catalytic activity of Sr_1-*x*_Na_*x*_RuO_3_ after 20 cycles with respect to the initial activity, including RSD as shadow bars

Figure 2a shows the current densities (*j*) normalized by the geometric area (0.196 cm^2^). Na-doped samples present similar current densities than SrRuO_3_ in the whole polarization range. The potential to reach a current density of *j* *=* 0.5 mA cm^−2^_geo_ is ~1.35 V for the three samples, equivalent to an overpotential (*η*) of only 120 mV. In comparison, the potential to achieve *j* *=* 0.5 mAcm^−2^_geo_ with RuO_2_ is ~1.4 V^16^. The low overpotentials of Sr_1-*x*_Na_*x*_RuO_3_ are in line with the potential reported to achieve the same current density for thin films of SrRuO_3_ in alkaline media of 1.35–1.40 V (the actual value depending on the exposed plane)^11^ and Ru metal in acid media (~1.32 V)^5^. However, the latter catalysts transform into RuO_*x*_ already in the first reaction cycle losing most of their initial activity.

As reported in Supplementary Table 7, the Na-containing perovskites have larger particle sizes than SrRuO_3_. Although it is well accepted that the OER is a structure sensitive reaction, the actual effect of particle size is not clear. Krtil et al.^24^ reported that the onset for the oxygen evolution current is particle-size independent for RuO_2_ electrodes, but not for Ru_0.8_Co_0.2_O_2−*x*_. By contrast, Reier et al.^25^ reported that nanosized Pt, Ru, and Ir particles are more active than the corresponding bulk phases. Shao–Horn et al.^26^ found that the OER activity of La_0.1_(Ba_0.5_Sr_0.5_)_0.9_Co_0.8_Fe_0.2_O_3−δ_ perovskites does not follow a linear relationship with particle size, and Schmidt et al.^27^ indicated that the OER activity of IrO_*x*_ does not increase monotonically with the surface area and suggest that morphology can also play a key role. In this sense, Matsumoto et al.^28,29^ reported that OER activity of mixed oxides could be related to the exposed facets, but failed to identify the most active ones. Recently, by using thin films of SrRuO_3_, Markovic et al.^11^ reported that the (111) face is more active for the OER than (001). Shao-Horn et al.^30^ reported that (100) facets of RuO_2_ and IrO_2_ are more active in alkaline media than the thermodynamically stable (110). We have found no evidences of preferential surface termination in our samples, but as shown in Supplementary Table 7, particle size increases with Na doping. Therefore, and in order to assess intrinsic catalytic activities (i_s_), the OER currents have been normalized to the oxide surface area, using BET, mass-specific surface areas (A_S_)^31^, or electrochemical surface areas (ECSA);^32,33^ see Supplementary Note 5 and Supplementary Table 7. The promotional effect of Na doping is clearly observed in Supplementary Fig. 8, where i_s_ follows the order Sr_0.90_Na_0.10_RuO_3 _≥ Sr_0.95_Na_0.05_RuO_3 _> SrRuO_3_. Recently, a protocol to account for the effect of bubbles in the active area during the OER has been reported^34^.

In Fig. 2b we report the OER intrinsic activities vs. A_S_. Na-doped perovskites show higher current densities than SrRuO_3_. For comparison, the potential to reach 10 mA cm^−2^_oxide_ with Sr_0.90_Na_0.10_RuO_3_ is 1.4 V; less positive than those reported for state-of-the-art perovskites of 1.5 V for SrIrO_3_/IrO_x_^15^ or 1.5–1.6 V for Ir-based double perovskites^14^. The actual production of O_2_ during the OER has been confirmed by using a RRDE (rotating ring disk electrode, see Supplementary Note 6 and Supplementary Fig. 9) and by following O_2_ evolution during the OER, using a mass spectrometer probe immersed in the electrolyte (Supplementary Note 7 and Supplementary Fig. 10).

Figure 2b compares the Tafel plots of the intrinsic activities of the catalysts under study with the best catalysts in acid electrolyte reported in the literature. The initial activities of Sr_1−*x*_Na_*x*_RuO_3_ are higher than the ones reported for similar catalysts. Currents similar to Ir double perovskites or SrIrO_3_/IrO_*x*_ (~5–10 mA cm^−2^_oxide_)^14,15^ are achieved by Sr_1-*x*_Na_*x*_RuO_3_ at significantly lower overpotentials.

The catalytic activity enhancement of SrRuO_3_ upon Na doping is analyzed in Fig. 2c using a DFT-based screening study in which the OER mechanism on all materials is assumed to proceed as: H_2_O → *OH → *O → *OOH → O_2_. This analysis outlines clear activity trends, namely that progressive Na doping weakens the adsorption energies because the surface oxygen p-band center and the Ru d-band center are positively displaced (see Supplementary Note 8 and Supplementary Fig. 11)^35^. The combinatorial model in Supplementary Note 9 and Supplementary Figs. 12 and 13 shows that doping is beneficial for moderate Na doping (0 < *x* < 0.125) but becomes deleterious at larger Na doping (*x* > 0.125); see Supplementary Figs. 12 and 13. Figure 2c shows that Sr_0.875_Na_0.125_RuO_3_ is at the top of the volcano and that its decomposition into RuO_2_ decreases the activity. The activity of various defective surfaces (Sr_0.875_RuO_3_, Sr_0.750_Na_0.125_RuO_3_, and Sr_0.625_Na_0.250_RuO_3_, RuO_x_/SrRuO_3_, see Supplementary Fig. 14c) is always lower than that of Sr_0.875_Na_0.125_RuO_3_ but generally higher than that of RuO_2_. These results show that as the pristine Na-doped perovskite decomposes (via Sr dissolution, Na dissolution or both, see Supplementary Note 10 and Supplementary Figs. 14b and 15), the activity decreases until it reaches that of RuO_2_, in agreement with our durability experiments (see below).

Although there is no one-to-one connection between theoretical and experimental overpotentials (Fig. 2b, c); Jaramillo et al.^36^ noted that the two overpotentials agree well when the experimental ones are taken at a current density of 1 mA·cm^−2^_oxide_. Thus, Fig. 2c contains suitable data from the literature showing good agreement between theory and experiments and confirms the high activity of Na-doped SrRuO_3_. The differences between Fig. 2b, c are probably due to DFT-based errors^37^, which in view of their intrinsic nature (because they are present in all data), translate into error bands around the volcano lines in Fig. 2c. In particular, the use of scaling relations (Supplementary Fig. 14a) and GGA exchange-correlation functionals set those errors at approximately ±0.2 eV for adsorption energies (±0.2 V for (over)potentials). See Supplementary Notes 11 and 12.

We stress here that screening analyses are not intended to elucidate the exact OER mechanism, which is the subject of different types of studies^38^, but rather to enable the simultaneous comparison of several different materials that clarify the role of Na incorporation and dissolution on the activity. Further discussion on this subject is provided in Supplementary Note 11. Note in passing that Govindarajan et al.^39^ recently proposed a descriptor called electrochemical-step symmetry index (ESSI) for OER catalysts (see Supplementary Note 11 and Supplementary Fig. 14b). Although the descriptor is based on the departures of OER reaction energies from the ideal value of 1.23 V, it does not depend on scaling relations, unlike the volcano plot in Fig. 2c. The conclusions drawn using ESSI are analogous to those of Fig. 2c, which substantiates our analysis. Furthermore, the ESSI analysis in Supplementary Fig. 14b suggests that Sr_0.875_Na_0.125_RuO_3_ could be further improved by strengthening its *OH adsorption energy by 0.16 eV.

### Stability during OER

Durability is a major concern when considering the actual implementation of an active OER catalyst in an electrolyzer, especially in acid electrolyte. As observed in Fig. 2d, replacing Sr by Na significantly improves the catalyst’s stability. After 20 consecutive cycles of reaction, SrRuO_3_ loses more than 85% of its initial activity. This is in line with the observation that SrRuO_3_ loses its activity after few cycles^11,13^. Remarkably, Na-doped perovskites only lose ca. 15% of their initial activity after 20 cycles. In other words, to lose 65% of initial activity, SrRuO_3_ needs only ~7 cycles while Sr_0.90_Na_0.10_RuO_3_ needs ~80 (Supplementary Note 13 and Supplementary Figs. 16 and 17). The voltammograms recorded during the OER (Supplementary Fig. 16) are in line with those of Ru-based perovskites^13^. Contrary to what is observed for Ir-based perovskites^40^, features due to the formation of oxides (RuO_x_) are not observed in the capacitive region of the voltammograms, probably because the expected features for the oxides overlap with the current due to the OER^13^. We emphasize the durability obtained for S_r0.95_Na_0.05_RuO_3_ and S_r0.90_Na_0.10_RuO_3_, first because most mixed oxides are not stable in acid, so finding a catalyst with such high activity, low overpotential, and durability over several cycles is already a noteworthy result. Second, because the high activities obtained are only met by Ru or Ir metals, which are rather unstable^41^. For instance, Ru metal in acid media loses most of its activity after the first cycle of reaction and completely dissolves within the first 10 cycles, due to metal oxidation when the potential rises^42^. RuO_2_ also presents high OER activity but suffers from corrosion after the first cycle; it should be noted, however, that RuO_2_'s performance for the OER is influenced by its morphology^43^. Finally, the number of perovskites reported as OER catalysts in acid is limited and those with high activity are SrIrO_3_ and SrRuO_3_. In SrIrO_3_, Sr dissolves after the first cycles and it is suggested that IrO_*x*_ oxides at the catalyst’s surface are responsible for the catalytic activity^15^. In SrRuO_3_, Sr dissolves decreasing the activity dramatically^11,13^. Durability studies of powder La_2_LiIrO_6_ in acid and alkaline conditions concluded that during the OER, IrO_2_ nanoparticles segregate to the perovskite surface, resulting in an oxidized surface with high OER activity^40^. In summary, most reports indicate that metal oxides are prone to severe degradation issues under oxygen evolution conditions^11,44^.

### Evolution of structure and composition during OER

Insights into deactivation of representative SrRuO_3_ and Sr_0.90_Na_0.10_RuO_3_ were obtained by analyzing fresh and used samples (after 20 OER cycles) and from the evolution of the composition of the electrolyte after 10, 40, and 80 OER cycles by ICP-OES (see also Supplementary Note 14).

**Fig. 3:**
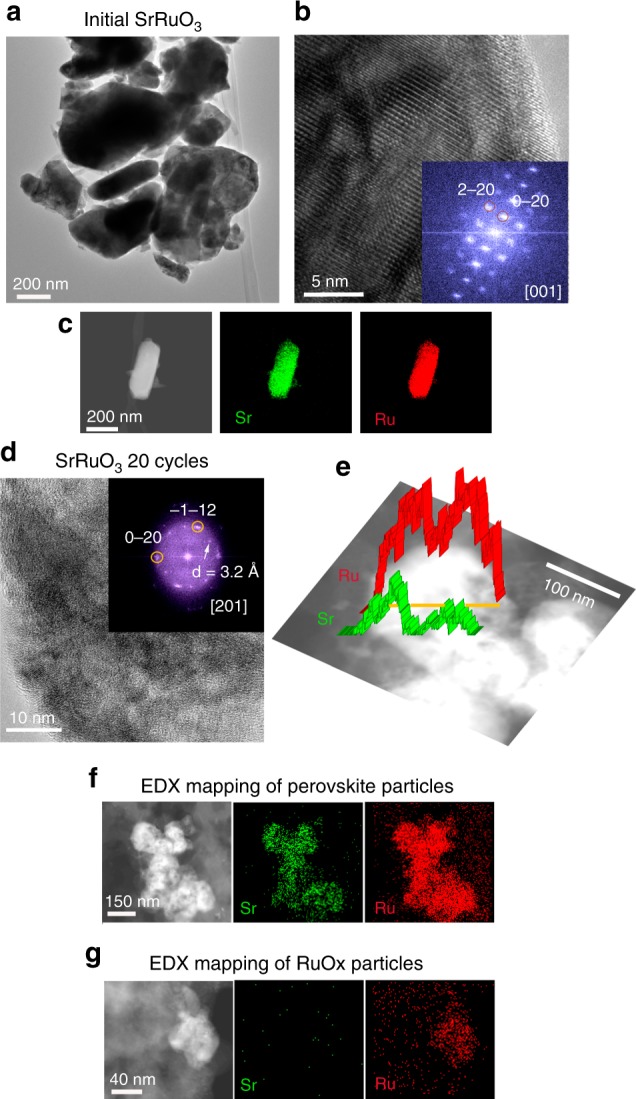
Evolution of SrRuO_3_ during OER. **a** TEM, **b** HRTEM/DDP and **c** STEM-EDX mapping of SrRuO_3_ perovskite before the OER reaction. **d** HRTEM/DDP, **e** STEM-HAADF micrographs with the line scans, **f** STEM-EDX mappings of SrRuO_3_ after 20 OER cycles, **g** STEM-EDX mapping of a RuO_*x*_ particle segregated after 20 OER cycles

As observed from the micrographs in Fig. 3a, SrRuO_3_ consists of particles between ca. 200 and 800 nm with perovskite structure, see HRTEM/Digital Diffraction Pattern (DDP) in Fig. 3b and HRTEM/SAED in Supplementary Fig. 4. STEM-EDX mappings (Fig. 3c) reveal a homogeneous distribution of Sr and Ru along the particle with an atomic Sr/Ru ratio close to 1. After 20 cycles in the OER, significant changes in the morphology and composition of SrRuO_3_ are observed. Figure 3d shows a HRTEM/DDP image of used SrRuO_3_. The DDP shows spots that can be ascribed to two different phases: the original perovskite in the [201] zone axis and spots of *d* = 3.2 Å attributed to the (110) interplanar distances in rutile RuO_2_. Compositional analyses of the used SrRuO_3_ (Fig. 3e–g) reveal the loss of Sr during the OER (voids are observed in the STEM image in Fig. 3e) leading to regions with high (~3) Ru/Sr ratio (Fig. 3e, f) along with segregated RuO_*x*_ particles as shown in Fig. 3g. This observation is consistent with the analysis of the composition of the electrolyte during OER with SrRuO_3_. As shown in Supplementary Fig. 17, the concentration of Sr in the electrolyte during OER cycles increases faster than that of Ru. HRTEM images in Supplementary Fig. 18 reveal an evident loss of crystallinity of used SrRuO_3_.

**Fig. 4:**
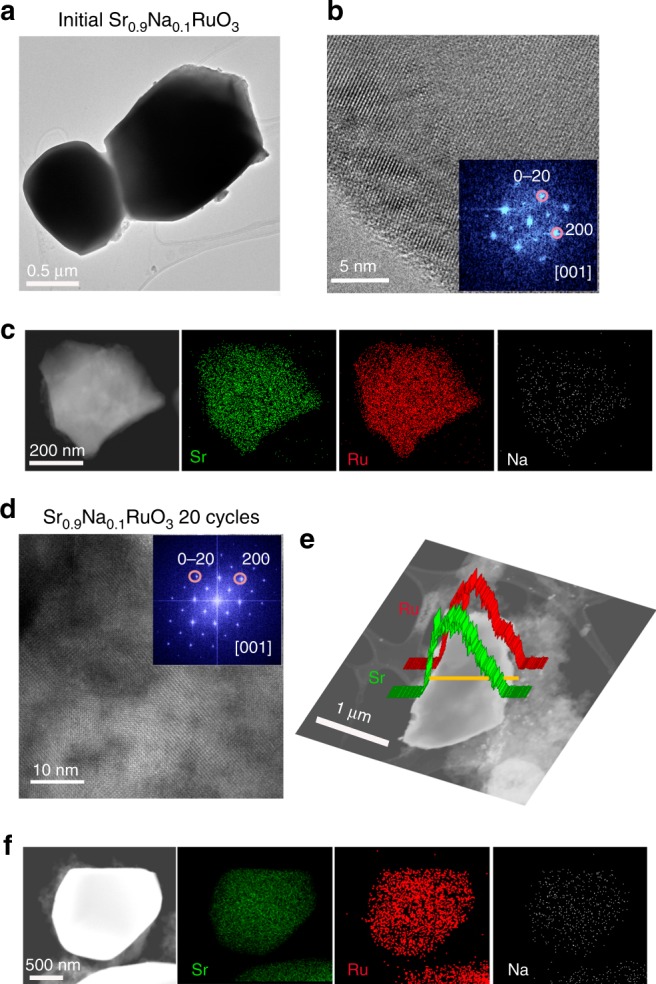
Evolution of Sr_0.90_Na_0.10_RuO_3_ during OER. **a** TEM, **b** HRTEM/DDP, and **c** STEM-EDX mapping of Sr_0.90_Na_0.10_RuO_3_ before the OER reaction. **d** HRTEM/DDP images, **e** STEM-HAADF micrographs showing Ru and Sr line scan profiles and **f** STEM-EDX mapping of Sr_0.90_Na_0.10_RuO_3_ after 20 OER cycles

Representative TEM and HRTEM/DDP images of Sr_0.90_Na_0.10_RuO_3_ are shown in Fig. 4a, b, respectively. The sample consists of particles of ~2.8 (±0.5) μm with perovskite structure; see DDP (inset of Fig. 4b and SAED in Supplementary Fig. 4). STEM-EDX mapping (Fig. 4c) reveals the homogeneous distribution of Sr, Ru, and Na in the perovskite. Figure 4d shows a representative HRTEM/DDP image for used Sr_0.90_Na_0.10_RuO_3_. Contrary to SrRuO_3_, used Sr_0.90_Na_0.10_RuO_3_ still presents the perovskite structure (inset of Fig. 4d). As shown in Fig. 4e, the size and Sr/Ru atomic ratio (~0.95) of used Sr_0.90_Na_0.10_RuO_3_ is similar to that of the fresh one. STEM-EDX mapping in Fig. 4f shows a homogeneous distribution of Sr, Ru, and Na in the used sample. These observations clearly indicate that neither the composition nor the structure of Sr_0.90_Na_0.10_RuO_3_ are affected after 20 cycles in the OER. HRTEM/DDP images (Supplementary Fig. 19) also reveal the high crystallinity of used Sr_0.90_Na_0.10_RuO_3_. However, note that the incipient formation of RuO_*x*_ is evidenced in the FFT filtered image shown in Supplementary Fig. 19b. Again, this observation is in good agreement with the ICP results shown in Supplementary Fig. 17b revealing small concentrations of Sr and Ru in the electrolyte after the OER, significantly lower than those found for SrRuO_3_. In fact, Supplementary Fig. 17b confirms that the Na-doped perovskite is more stable than SrRuO_3_ during the OER in acid electrolyte.

**Fig. 5:**
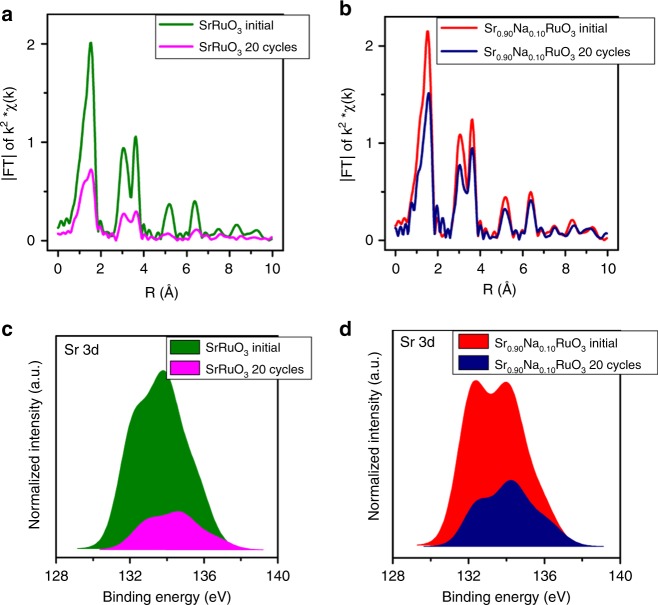
XAS and XPS of fresh and cycled samples. **a** Fourier transform from Ru K-edge EXAFS signal before and after OER of SrRuO_3_ and **b** of Sr_0.90_Na_0.10_RuO_3_. **c** Sr-3d core-level region before and after OER of SrRuO_3_ and **d** of Sr_0.90_Na_0.10_RuO_3_. The intensity of the Sr peaks in the used samples is normalized to the intensity of Sr in the fresh samples

The structure of the used samples (20 cycles in the OER) has also been studied by XRD and XAS. The x-ray diffractograms of the fresh and used samples are similar and only show diffraction lines for the perovskite phase (Supplementary Fig. 20). The analysis of the Ru K-edge of fresh and used SrRuO_3_ and Sr_0.90_Na_0.10_RuO_3_ are shown in Fig. 5 and Supplementary Fig. 21. The intensity of the FT-EXAFS signals of the used SrRuO_3_ sample is significantly lower than that of the fresh one, especially for the higher shells (Fig. 5a). This result reveals severe degradation (loss of crystallinity) of the SrRuO_3_ structure after the OER. Conversely, the FT-EXAFS signals of both fresh and used Sr_0.90_Na_0.10_RuO_3_ are similar (Fig. 5b), indicating that the perovskite structure remains stable during the OER. It is possible to estimate the composition of the used samples by a linear combination fit on the EXAFS signals of the fresh perovskites and RuO_2_. An approximate ratio of 20:80 SrRuO_3_:RuO_2_ is obtained for SrRuO_3_ (Supplementary Fig. 21c) being of 80:20 Sr_0.90_Na_0.10_RuO_3_:RuO_2_ for the Na-doped one (Supplementary Fig. 21d). These results are in excellent agreement with the microscopy results, and indicate that Na doping enhances the structural stability (and hence durability) of the perovskites during the OER. These conclusions also result from the evolution of the pre-edge features of the XANES Ru K-edge and the decrease in the white line (Supplementary Fig. 21a).

Finally, the surface composition of the fresh and used perovskites was analyzed by XPS. Figures 5c, d depict the spectra of the Sr 3d core-level regions of the fresh and used samples. The two Sr 3d doublets are associated to carbonate (high binging energy) and Sr–O–M (lower binding energy) moieties. According to Supplementary Table 8, the surface atomic Ru/Sr ratio of both SrRuO_3_ and Sr_0.90_Na_0.10_RuO_3_ increases after the OER. Nevertheless, Ru enrichment is more pronounced in SrRuO_3_ (2.7) than in Sr_0.90_Na_0.10_RuO_3_ (1.8) indicating a more severe decomposition of SrRuO_3_.

The results above clearly indicate that Sr dissolves from the perovskite structure during the OER, especially from SrRuO_3_. Upon Sr dissolution, the perovskite structure becomes ill-defined, as observed by TEM and XAS. At some point, Ru segregates from the perovskite and forms nanosized RuO_*x*_ phases, which in some cases are deposited at the surface of the perovskites, but are mostly found as isolated particles (Supplementary Fig. 18). As predicted by DFT (Fig. 2c), the collapse of the perovskite and the formation of RuO_*x*_ lead to a significant loss in OER activity. The higher durability of Na-doped samples is due to their higher structural stability and lower dissolution rates of Sr and Ru compared with SrRuO_3_, not to the formation of different phases during the OER. Several features can explain the enhanced durability of Na-doped perovskites. On the one hand, it is known that Ru centers oxidize to Ru^>4+^ during potential excursions above ~1.4 V^45^. These highly oxidized Ru atoms are not stable within undoped SrRuO_3_^9^. Na incorporation permits the stabilization of Ru^>4+^ ions within the lattice, resulting in versatile Ru catalytic centers that can easily increase or decrease their charge depending on the adsorbates, which is advantageous for the OER. Our results show that Na incorporation results in less distorted RuO_6_ octahedra and higher durability. This observation is in line with previous reports indicating that less distorted structures are more susceptible to suffer structural variations without being destroyed when subjected to voltage changes^6^. Finally, DFT calculations indicate that (i) the surface energies of Sr_1−*x*_Na_*x*_RuO_3_ are considerably lower compared with SrRuO_3_; (ii) the dissolution potentials of the cations in the A site of the perovskites are shifted toward more positive values in presence of Na; and (iii) the stability at low pH is also enhanced upon Na doping. Such observations, described in detail in the Supplementary Note 10, attest to more stable perovskite structures upon doping.

To summarize and conclude, we have synthesized a series of active catalysts (Sr_1−*x*_Na_*x*_RuO_3_) for the OER in acid media with extraordinary features. The measured activities are comparable to or surpass those of other remarkable OER catalysts in the literature. Na incorporation in the lattice also grants these compounds high electrochemical and structural stability, so as to keep up to 85% of their initial activity after 20 cycles. Na locally changes the oxidation state of Ru from +4 to +5, resulting in a convenient weakening of the adsorption energies of the OER intermediates, because SrRuO_3_ binds those too strongly. Our conclusions could help in guiding future research in several ways: (a) other monovalent cations such as Li^+^, K^+^, and Cs^+^ can be tested to ascertain whether they also enhance the activity and/or the stability. (b) If Ru^5+^ sites are instrumental for the enhancement of SrRuO_3_, then B-site doping with e.g., trivalent cations might as well enhance the activity. (c) Since Sr_0.875_Na_0.125_RuO_3_ is the most active compound in Fig. 2c, we used the ESSI to determine that its activity can be further optimized if the *OH binding energy is strengthened by −0.16 eV. This is an interesting hypothesis to be verified experimentally in subsequent studies.

## Methods

### Synthesis

Sr_1-*x*_Na_*x*_RuO_3_ (*x* = 0.00, 0.05, 0.10) perovskites were synthesized in polycrystalline form by a wet-chemistry procedure to obtain very reactive precursors and reduce the final synthesis temperature. Stoichiometric amounts of Sr(NO_3_)_2_ (Sigma Aldrich 99.99% trace metals basis), Na_2_CO_3_ anhydrous (Sigma Aldrich 99.999% trace metals basis), and RuO_2_ (Sigma Aldrich 99.9% trace metals basis) were dissolved in 150 mL of 0.1 M citric acid (Sigma Aldrich assay ≥ 99.5%) and 20 mL of nitric acid (Panreac 65%), to ensure complete dissolution of the starting materials. We added 5% excess of Na_2_CO_3_ to compensate the possible partial evaporation at high temperatures. The mixture was stirred at ~120 °C until complete evaporation, leading to the formation of resins containing a homogeneous distribution of the metal cations. The resins were dried afterwards at 500 °C for 12 h, to eliminate organic material and nitrates. Finally, the samples were subjected to different temperatures in air during 12 h: 800 °C for *x* = 0.00, 900 °C for *x* = 0.05 and 1000 °C for *x* = 0.10. If SrRuO_3_ is calcined at higher temperatures, other competitive phases appear.

### Inductively coupled plasma optical emission spectrometry (ICP-OES)

An ICP-OES Analytik Jena PQ 9000 spectrometer was used for the analyses. Specimens for analysis were subjected to acid digestion in a mixture of 3 mL of HNO_3_, 2 mL HCl, 3 mL HF, and 3 mL H_3_PO_4_, in a pressurized microwave.

### Powder X-ray diffraction (XRD)

The phases and their purity were determined by XRD in Bragg–Brentano reflection geometry with CuK_α_ radiation (*λ* = 1.5418 Å).

### Powder neutron diffraction (PND)

PND was carried out in the high-resolution powder diffractometer D2B at ILL (Grenoble, France). The data were collected at room temperature and *λ* = 1.594 Å. For the refinement of the crystal structures, we used the Rietveld method and the Fullprof crystallographic program^46,47^. The function selected to generate the diffraction peaks shape was pseudo-Voigt. The parameters refined in the final run were the scale factor, linear interpolation between a set of background points, zero-point error, pseudo-Voigt parameters, positional coordinates, isotropic thermal factors an occupancy factors.

### Transmission electron microscopy (TEM)

TEM data was recorded on a JEOL 2100 field emission gun transmission electron microscope operating at 200 kV and equipped with an EDX spectrometer Oxford INCA Energy 2000 system. Specimens were prepared by depositing small portions of the samples on top of a Cu grid supporting a lacey carbon film. Deposition was achieved by preparing a suspension of the material in ethanol.

### BET method

The measurements were performed in a Micromeritics ASAP 2000 apparatus. Surface areas were evaluated by purging with nitrogen the samples within the relative pressure range *P*/*P*_0_ = 0.05–0.30. The amount of adsorbed nitrogen is related to the total surface area of the samples. The volume of gas adsorbed on the surface is measured at −196 °C (nitrogen boiling point). The samples are finally degassed at 140 °C under vacuum for 24 h. Specific areas were calculated by applying the BET method.

### X-Ray absorption spectroscopy (XAS)

XAS measurements were performed at room temperature at Diamond Light Source (UK) on the B18 and I10 beamlines^48^. In the case of I10 measurements, data were collected at Na K-edge (*E* = 1070.8 eV) and Ru M_2,3_-edge (*E* = 461 and 483 eV) in fluorescence mode with a photodiode sited to measure by back-scattering geometry. The Sr L3 and L2-edges appear at the half energy (1940 eV and 2007 eV, respectively) as the undulator second harmonic is mostly absorbed but it partially passes through the monochromator at the energy of the first harmonic as a contamination. On the B18 beamline, data were collected at Ru K-edge (*E* = 22117 eV) in the transmission mode using a double crystal Si111 monochromator and Pt-coated branch. Data treatment was performed with the ATHENA software^49^ and XANES simulations were performed with the FEFF 8.4 code^50^.

### X-Ray photoemission spectroscopy (XPS)

X-ray photoelectron spectra (XPS) were acquired with a VG ESCALAB 200 R at a pass energy of 50 eV using a Mg Kα X-ray source. The kinetic energies of the photoelectrons were measured using a hemispherical electron analyzer working in the constant-pass energy mode. The background pressure in the analysis chamber was kept below 3 × 10^–8^ mbar during data acquisition. At least 200 scans were collected in increments of 0.1 eV with dwell times of 50 ms in order to increase the signal-to-noise ratio. The binding energies (±0.2 eV) were determined by setting the C 1s peak at 284.8 eV.

### Electrochemical characterization

The electrochemical performance was tested in a computer-controlled Autolab PGstat 302 N potentiostat/galvanostat. A standard three-compartment glass cell and a rotating disk electrode (RDE) (Pine Research Instruments) were used. A graphite rod and a homemade Reversible Hydrogen Electrode (RHE) were used as counter and reference electrodes, respectively. Sr_1−*x*_Na_*x*_RuO_3_ samples were deposited on the electrode as inks. The powdered samples were mixed in a 5:1 mass ratio with carbon black (Vulcan-XC-72R) to improve the electrical conductivity. Then the solvents were added, Tetrahydrofuran (THF) and Nafion, to yield final concentrations of 5 mg_oxide_ mL^−1^_ink_, 1 mg_carbon_ mL^−1^_ink_, 0.03 mL_Nafion_ mL^−1^_ink_, and 0.97 mL_THF_ mL^−1^_ink_. We sonicated the ink with an Ultrasonic Processor UP50H (Hielscher) and dispersed 20 μL of ink onto a glassy carbon electrode (0.196 cm^2^ area), with a final loading of catalyst on the electrode of 0.1 mg. We performed cyclic voltammetry between 1.1 V and 1.5 V or 1.1 V and 1.7 V at 10 mV/s and 1 mV/s. The measurements were corrected under O_2_ saturated electrolyte to assure the O_2_/H_2_O equilibrium at 1.23 V, and with an electrode rotation of 1600 rpm. The OER kinetic curves were capacitance-corrected by taking the average of the anodic and cathodic curves. The curves were also iR-corrected using the formula E-*iR*_corrected_ = E_applied_–*iR*, where *i* is the current and *R* is the ohmic electrolyte resistance (*R* ~29 Ω) obtained from Electrical Impedance Spectroscopy (EIS) at open voltage. The OER electrocatalytic measurement methodology follows the one established by Shao-Horn and coworkers^7,23^. As stated above, we used a catalyst loading of 0.1 mg, the same reported in previous works. Such high loading could result in film diffusion resistance that prevents the determination of kinetic parameters from non-corrected K-L analyses^51^. However, the absence of mass transfer effects has been confirmed by conducting the OER at different scan and rotation rates, and by using different catalyst loadings, as detailed in Supplementary Note 15 and Supplementary Fig. 22. We have measured catalytic activity and durability at least five times for each catalyst. Errors are reported as relative standard deviation (RSD).

### Computational methods

The spin-unrestricted DFT calculations of Sr_*X*_Na_1−*x*_RuO_3_ were performed with VASP^52^, using the RPBE exchange-correlation functional^53^, the projector augmented wave (PAW) method^54^, and Dudarev’s DFT + U formalism^55^. The simulated slabs were 4-layer thick: the atoms in the two topmost layers and the adsorbates could move in all directions, whereas those in the two bottommost layers were fixed at the converged bulk distances. The relaxations were performed with the conjugate-gradient scheme, using as convergence criterion for the ionic loops a maximal force on any atom of 0.05 eV Å^−1^. We used 2 × 2 (001) perovskite slabs, which contained eight SrRuO_3_ formula units. When one of the Sr atoms was replaced by Na, the slab was Sr_7_Na_1_Ru_8_O_24_, equivalent to Sr_0.875_Na_0.125_RuO_3_. We used a 4 × 4 × 1 k-point mesh and a plane-wave cutoff of 400 eV to ensure convergence of the adsorption energies within 0.05 eV. We added ~15 Å of vacuum between periodical images in the *z* direction and applied dipole corrections. We used Gaussian smearing with k_B_T = 0.2 eV, and extrapolated all energies to 0 K. The gas-phase molecules (H_2_, H_2_O) were calculated in boxes of 15 Å × 15 Å × 15 Å, with k_B_T = 0.001 eV and a 1 × 1 × 1 k-point mesh. The value of U for Ru was 6.7 eV, found by Kitchin and coworkers for RuO_2_ through a linear-response methodology^56^. Such value is applicable to the Ru perovskites under study in view of the identical oxidation state of Ru (+4) in RuO_2_ and SrRuO_3_ and its similar coordination to oxygen in the form of octahedral RuO_6_ complexes. RuO_2_ in Fig. 2c was also calculated using the DFT + U formalism with the same value of U. Supplementary Note 12 discusses the inaccuracies of standard DFT for the evaluation of adsorption energies of RuO_2_(110)^56^ and plausible solutions^56,57^. The lattice constant found for SrRuO_3_ was 4.033 Å and that of Sr_0.875_Na_0.125_RuO_3_ was 4.018 Å. As a first approximation, the former was used for all slab calculations. The free energies were approximated as: G = E_DFT_ + ZPE−TS, where E_DFT_ and ZPE are the DFT total and zero-point electronic energies, and TS are entropic contributions (only taken into account for gas-phase species). The TS corrections for H_2_ and H_2_O_(l)_ are 0.40 and 0.67 eV^58,59^. The ZPEs for H_2_, H_2_O, *O, *OH and *OOH are 0.27, 0.56, 0.07, 0.35, and 0.40 eV, respectively. To describe the energetics of (H^+^ + e^−^) and estimate overpotentials we used the computational hydrogen electrode^58^. The procedure for estimating the free energies of adsorption of the oxygen evolution intermediates, and details of the construction of the volcano plots making use of the scaling relations between the adsorption energies of the adsorbates (see Supplementary Note 11) appear elsewhere^58,60,61^. We included total adsorbate coverages of 0.5 ML in all cases, so that *O, *OH, and *OOH were co-adsorbed with *O. Such co-adsorption accounts for the fact that at OER potentials at least part of the active Ru sites are likely covered with *O. The assessment of the ESSI is described in the Supplementary Note 11 and elsewhere^39^.

## Supplementary information

Supplementary Information

## Data Availability

The data that support the findings of this study are available within the article and its Supplementary Information files. All other relevant data supporting the findings of this study are available from the corresponding authors upon request.
