# Supplementary material for: Na-doped ruthenium perovskite electrocatalysts with improved oxygen evolution activity and durability in acidic media
Source: Nat Commun. 2019 May 3;10:2041. doi: 10.1038/s41467-019-09791-w (PMC6499887; doi:10.1038/s41467-019-09791-w)
Supplement: Supplementary file 1 — Supplementary Information [file 41467_2019_9791_MOESM1_ESM.pdf]

Supporting Information for:

Na-doped Ruthenium Perovskite Electrocatalysts with Improved Oxygen Evolution Activity and Durability in Acidic Media

Retuerto et al.

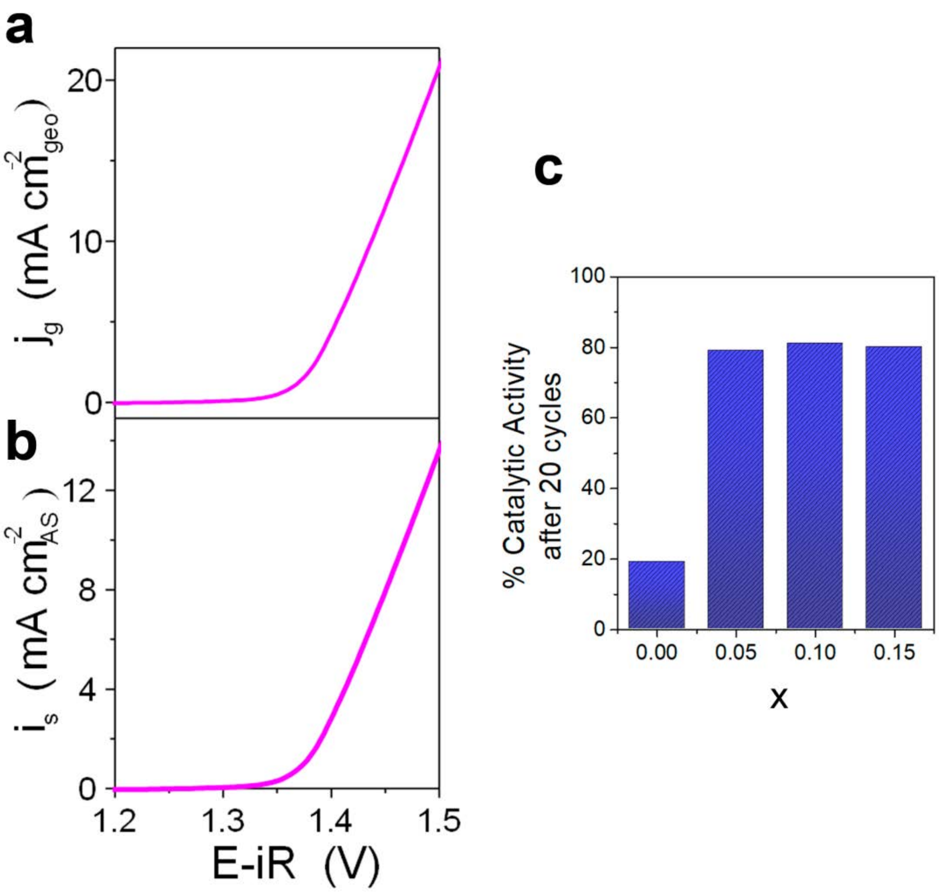

Supplementary Figure 1. Catalytic activity of $Sr_{0.85}Na_{0.15}RuO_3$. **a** Current density. **b** Intrinsic activity. **c** Percentage of the catalytic activity of $Sr_{1-x}Na_xRuO_3$ after 20 cycles with respect to the initial activity.

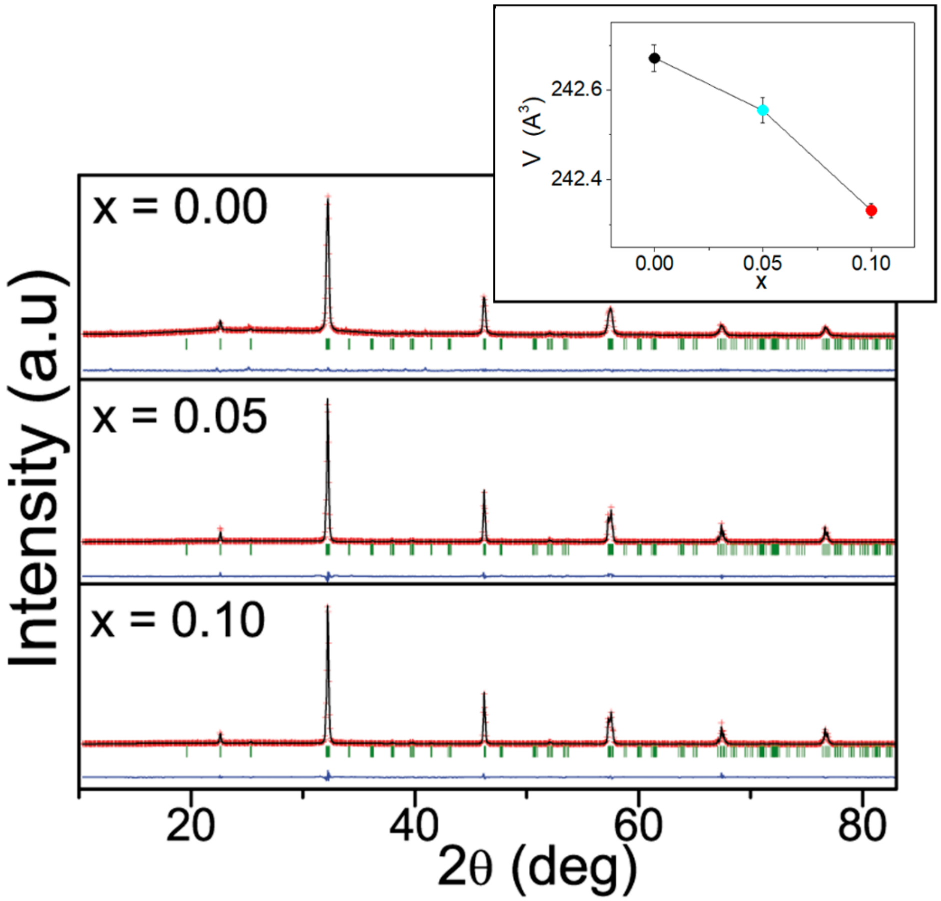

Supplementary Figure 2. Room temperature XRD patterns of $Sr_{1-x}Na_xRuO_3$. Rietveld refinements of their crystal structures; XRD spectrum (black line), fitted spectra (red) and residue (blue line). Green lines are the Bragg reflections. Inset: Cell volume evolution.

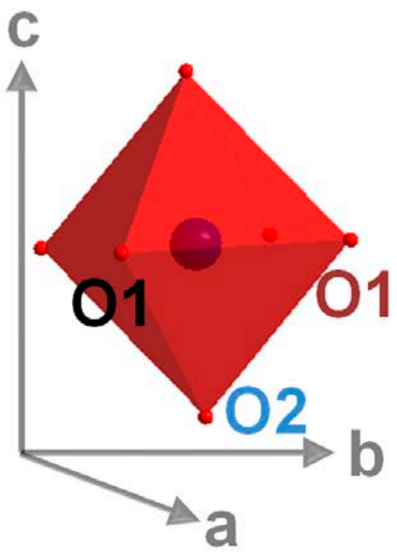

Supplementary Figure 3. Schematic view of RuO_6 octahedra with oxygen atoms labelled as O1 and O2.

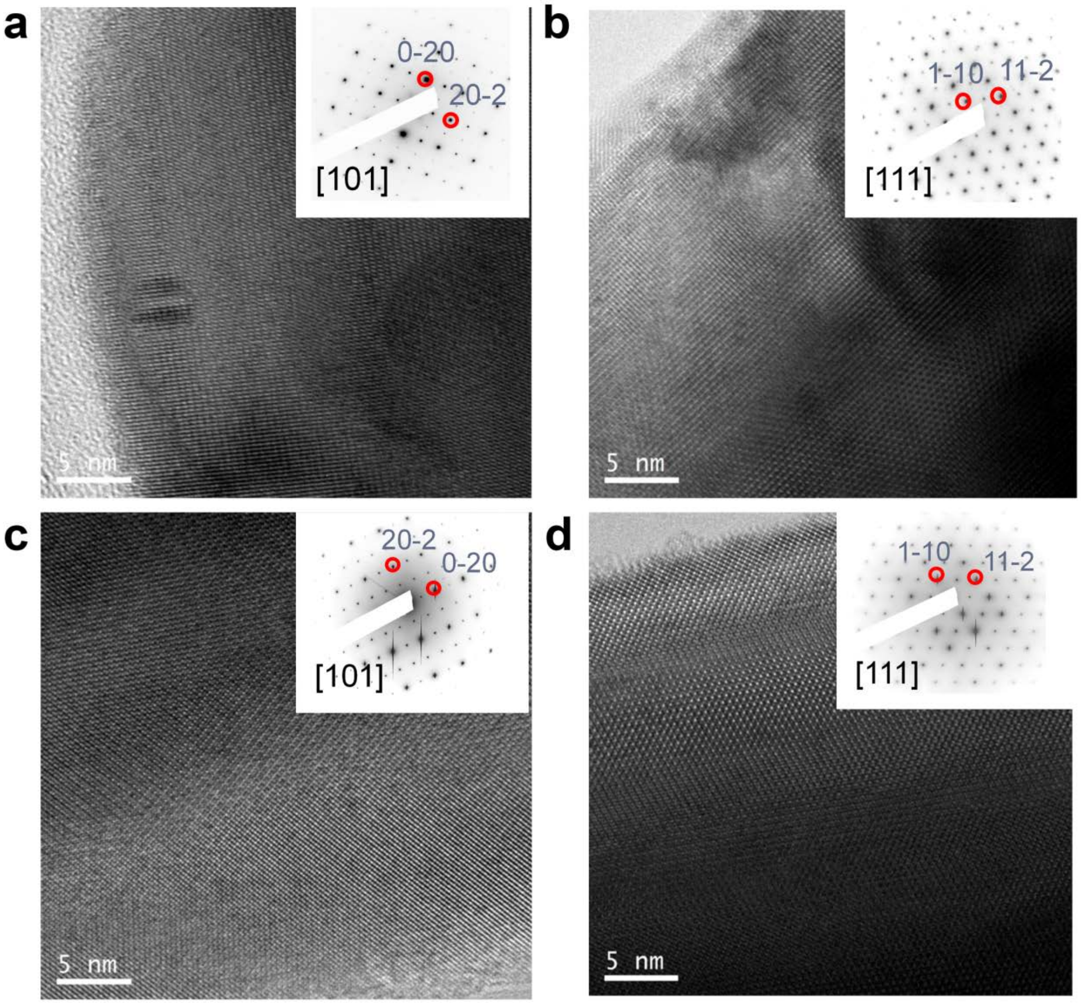

Supplementary Figure 4. HRTEM/SAED images of $SrRuO_3$ in **a** [101] zone axes and **b** [111] zone axes. HRTEM/SAED images of $Sr_{0.90}Na_{0.10}RuO_3$ in **c** [101] zone axes and **d** [111] zone axes.

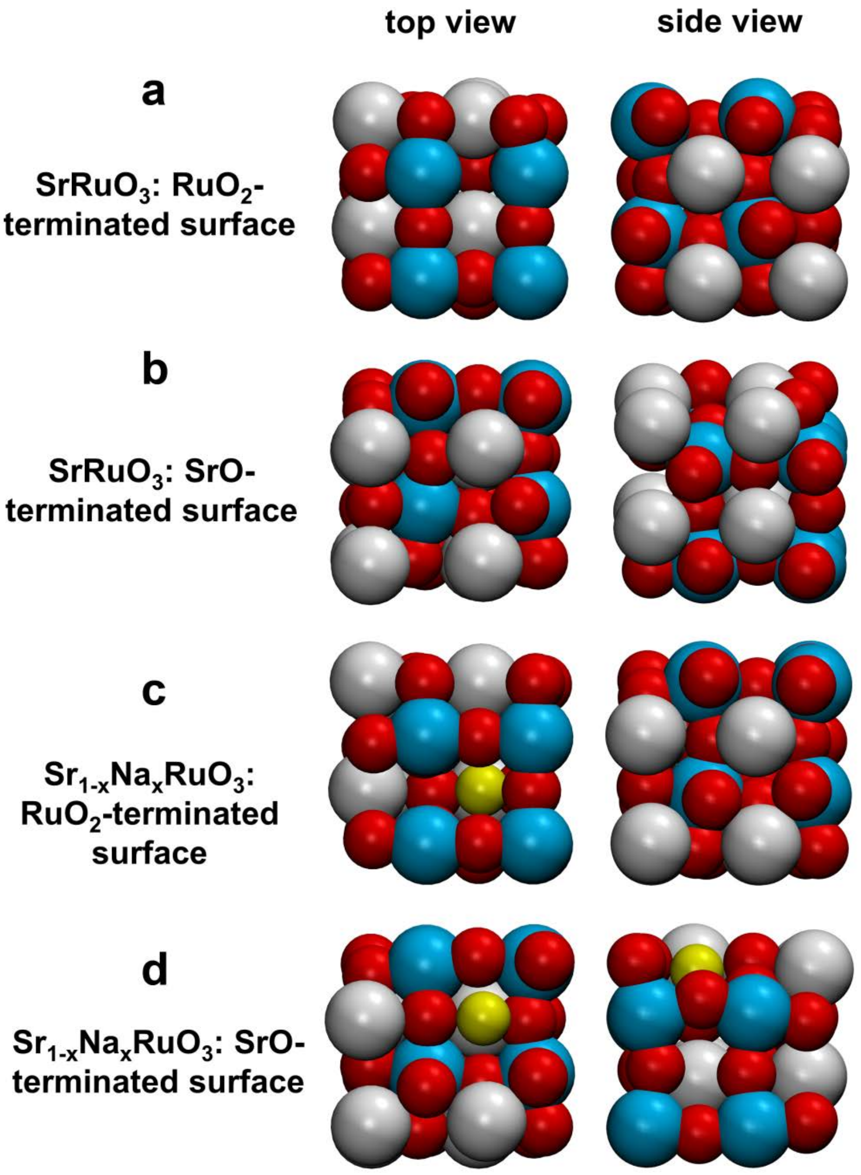

Supplementary Figure 5. Top and side view of the units used to calculate the oxygen vacancies formation energies reported in Supplementary Table 6. **a** RuO_2-terminated surface on $SrRuO_3$. **b** SrO-terminated surface on $SrRuO_3$. **c** RuO_2-terminated surface on $Sr_{1-x}Na_xRuO_3$. **d** SrO-terminated surface on $Sr_{1-x}Na_xRuO_3$. White: Sr, yellow: Na, blue: Ru, red: O.

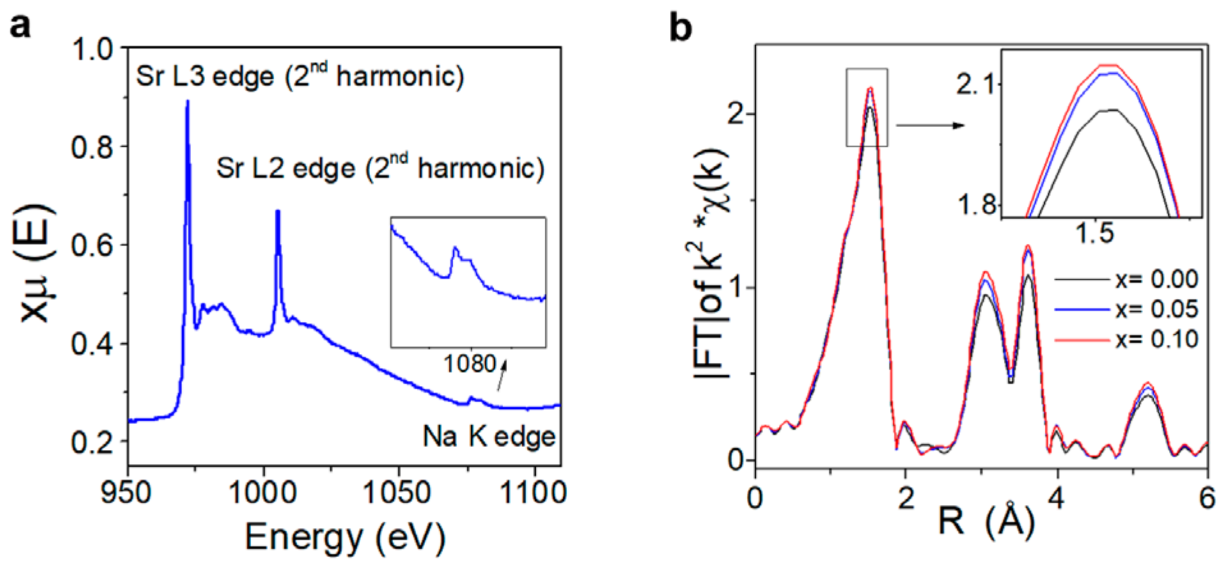

Supplementary Figure 6. X-ray Absorption Spectroscopy. **a** Sr L3, Sr L2 and Na K-edges XANES spectra of $Sr_{0.95}Na_{0.05}RuO_3$. **b** Fourier Transformation from Ru K-edge EXAFS signal for $Sr_{1-x}Na_xRuO_3$ (x= 0.00, 0.05, 0.10).

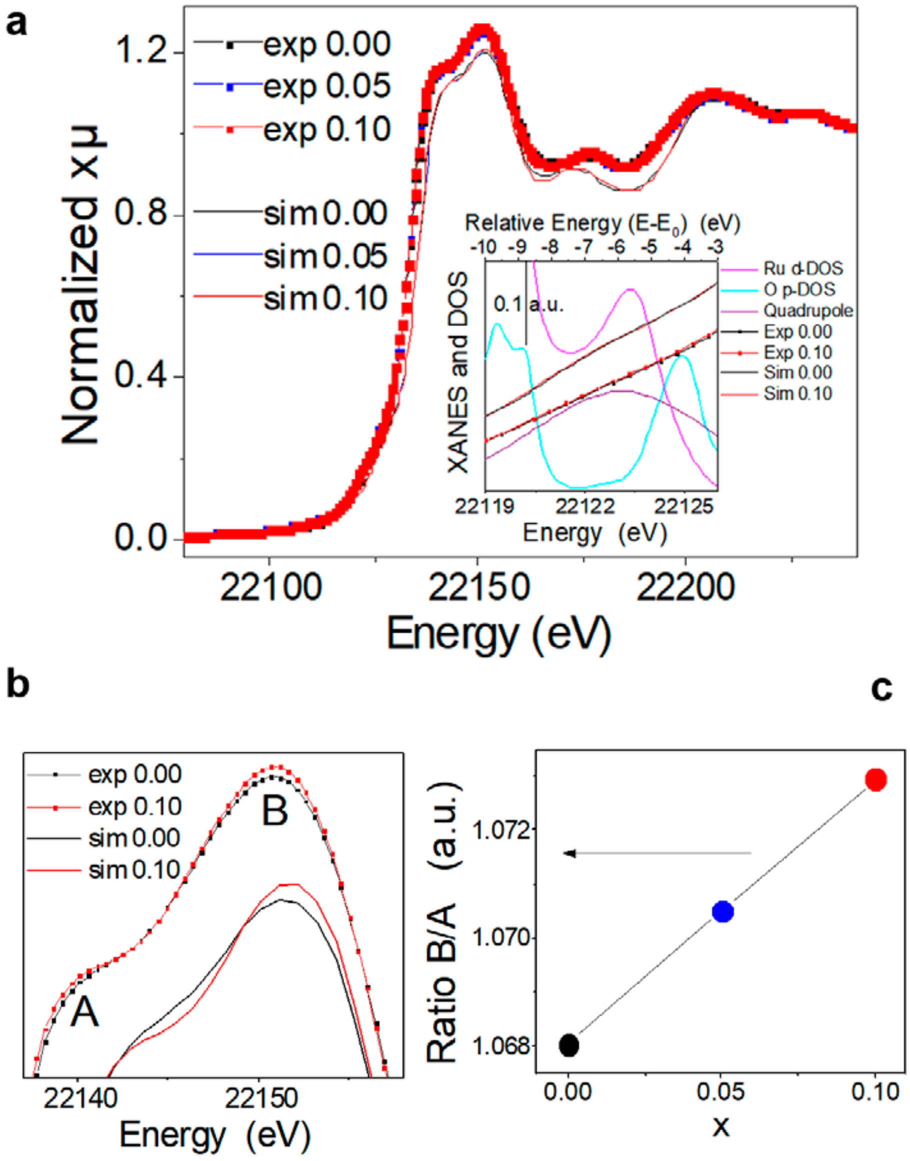

Supplementary Figure 7. X-ray Absorption Spectroscopy. **a** Experimental and theoretical calculations of Ru K-edge XANES spectra of $Sr_{1-x}Na_xRuO_3$ (x=0.00, 0.05, 0.10). Inset: Analysis of the pre-edge. **b** Magnification of the two main peaks after the edge (22140 and 22151 eV), experimental and simulation. **c** Comparison of the B/A intensity ratios with Na doping Powder Neutron Diffraction (PND) levels.

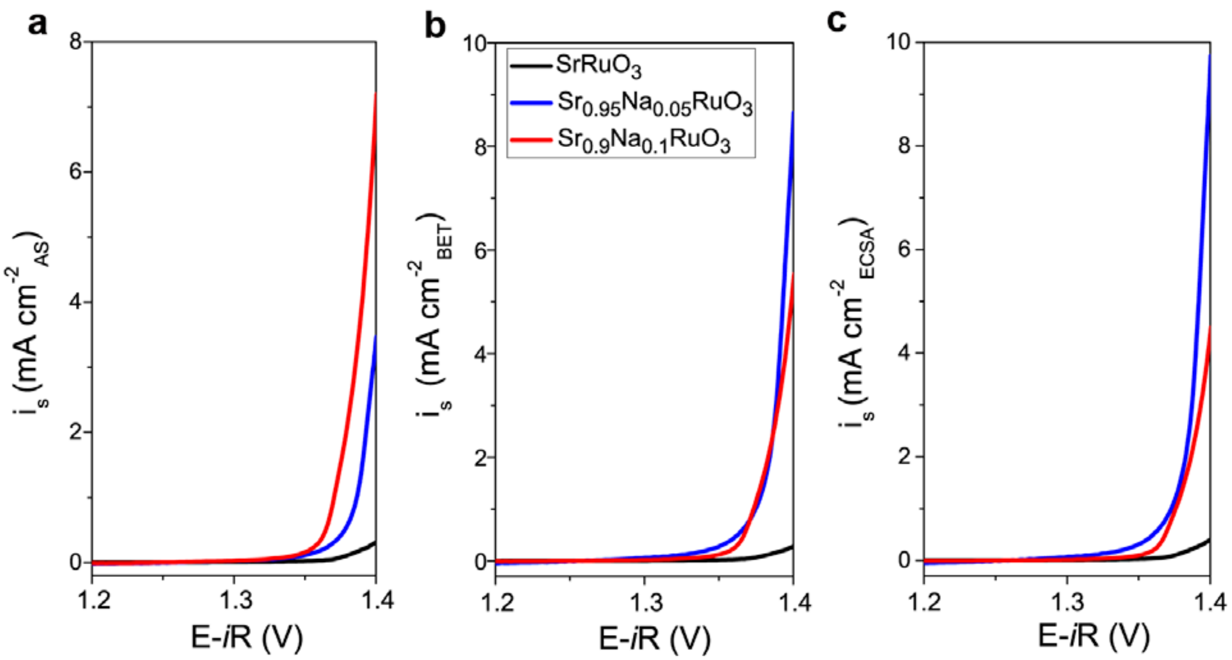

Supplementary Figure 8. Intrinsic activities for $Sr_{1-x}Na_xRuO_3$ (x= 0.00, 0.05, 0.10) measured in 0.1 M $HClO_4$, 1 mVs^{-1} and 1600 rpm. Activity normalized by **a** A_S, **b** BET and **c** ECSA.

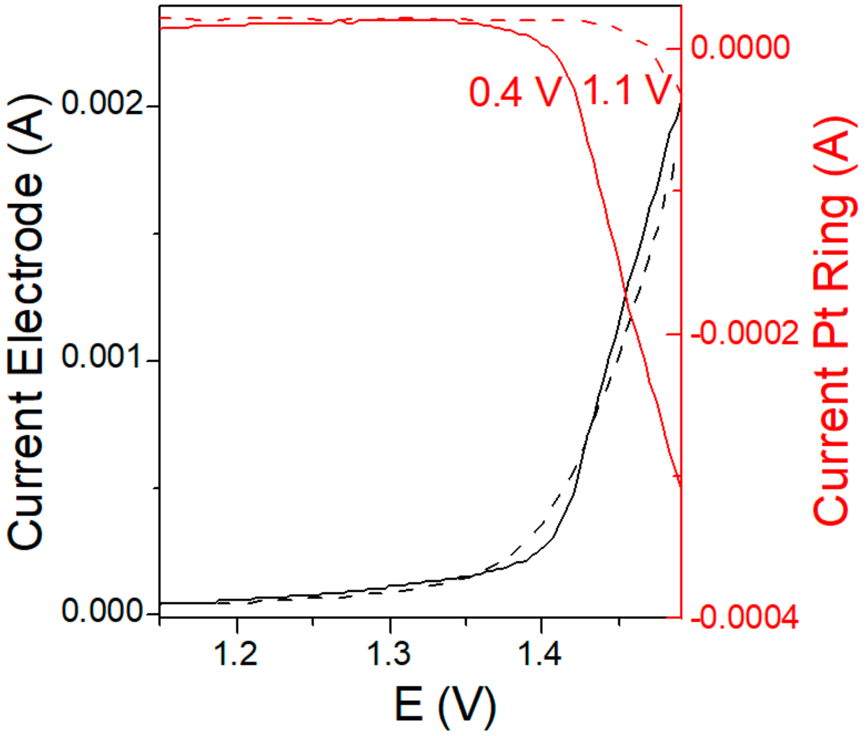

Supplementary Figure 9. RRDE experiments recorded with $Sr_{0.90}Na_{0.10}RuO_3$ during the positive going scan between 1.1 and 1.5 V in 0.1 M $HClO_4$ at 10 mVs^{-1}. Dotted lines are for the experiment recorded by setting the ring at 1.1 V, whereas straight lines correspond to the experiment recorded with the ring at 0.4 V.

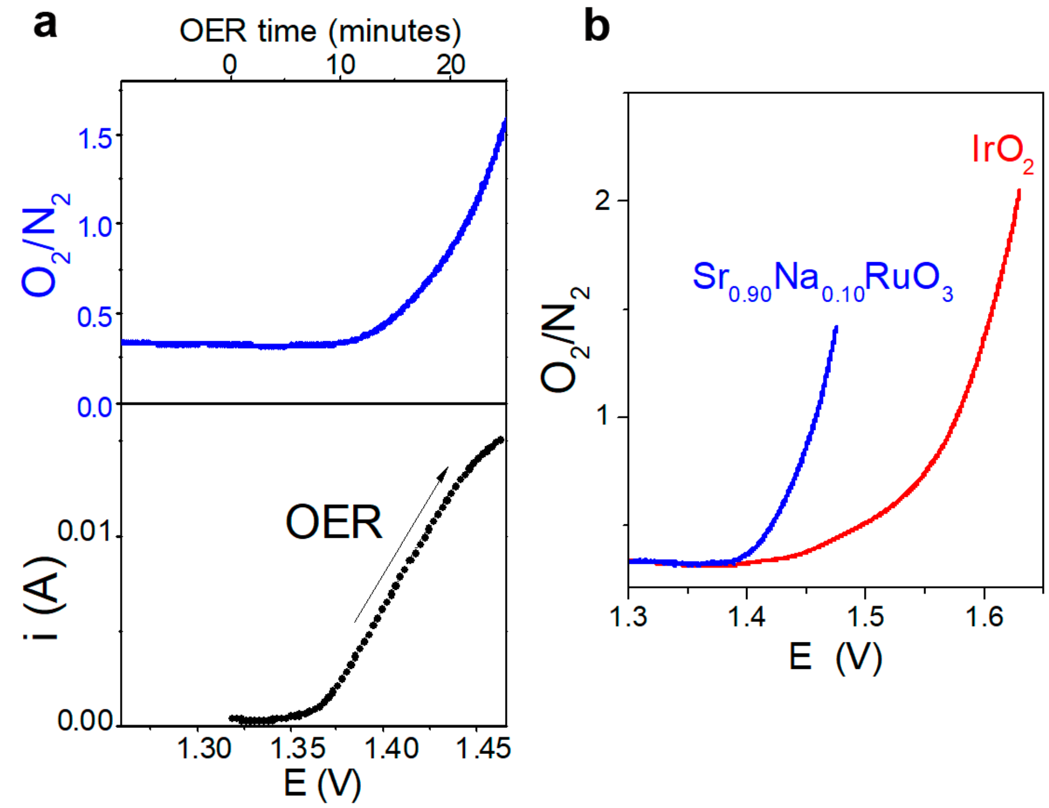

Supplementary Figure 10. *In situ* Mass Spectrometry (MS) measurements. **a** Upper panel, O_2 (m/z 32)/N_2 (m/z 28) mass signal ratio obtained from MS collected close to the working electrode during a OER linear single scan (LSV) between 1.3 and 1.5 V at 0.1 mVs^{-1} in Ar-purged 0.1 M $HClO_4$ (lower panel). **b** O_2/N_2 mass signal ratio recorded during an OER measurement (LSV) for $Sr_{0.90}Na_{0.10}RuO_3$ (blue) and IrO_2 (red).

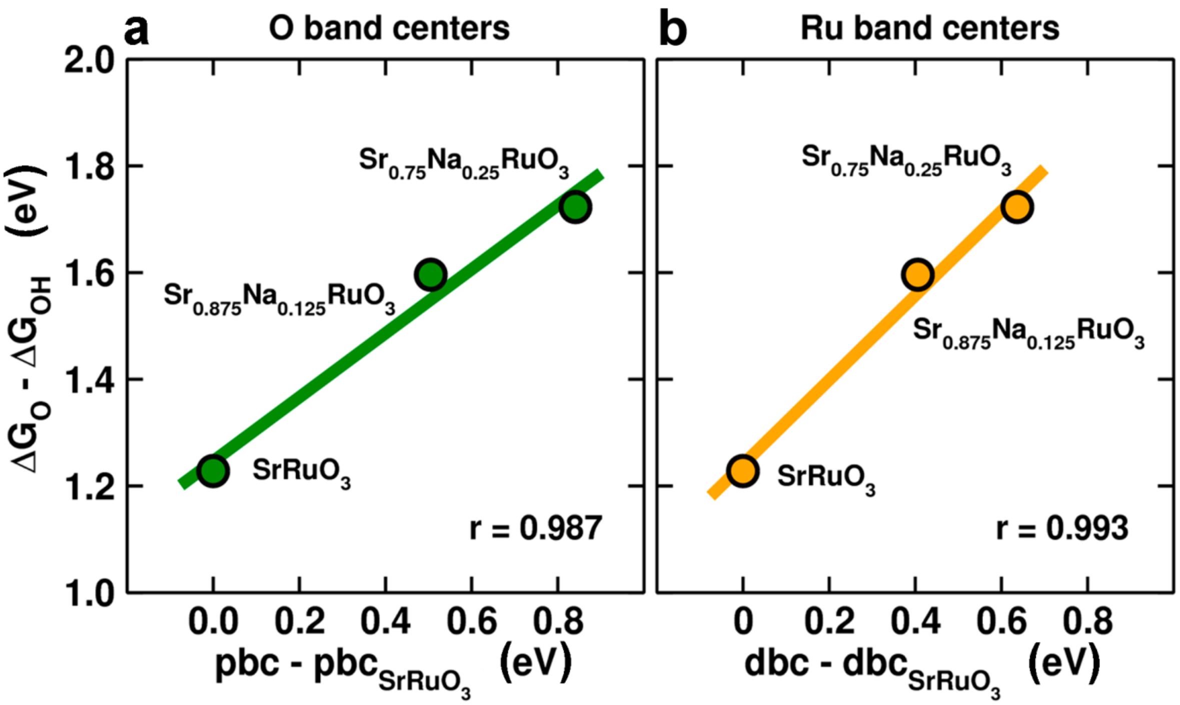

Supplementary Figure 11. Variations in band centers and adsorption energies in pure and Na-doped $SrRuO_3$. **a** O p-band centers. **b** Ru d-band centers.

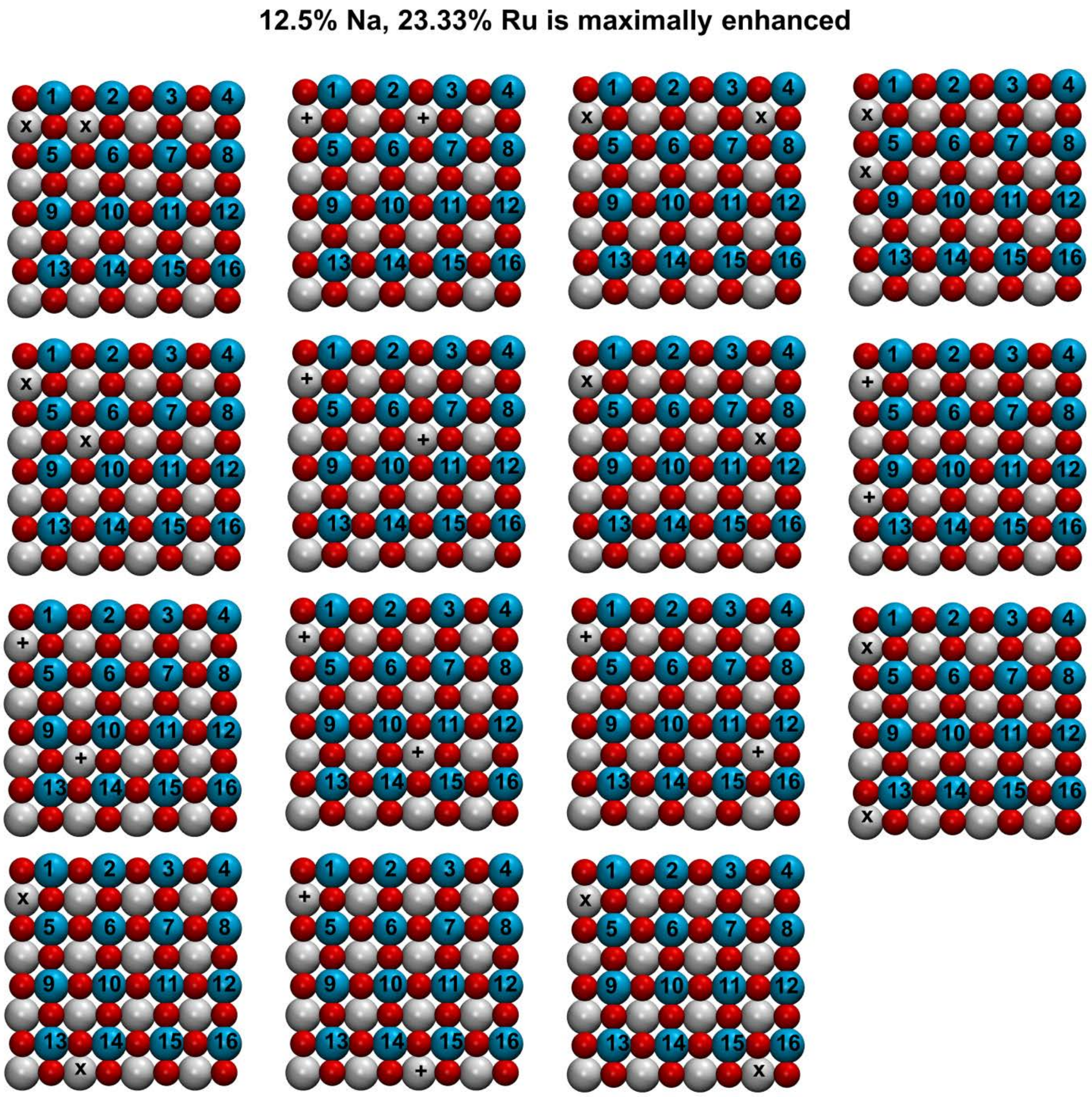

Supplementary Figure 12. Top views of 4×4 $SrRuO_3$ (100). Ru: blue, O: red, Na/Sr: white. Ru ions have been enumerated and Na atoms are marked with crosses (x, when the configuration leads to Ru sites with more than 2 Na adjacent to it) or plus signs (+, when the configuration leads to Ru sites with no more than 1 Na adjacent to it).

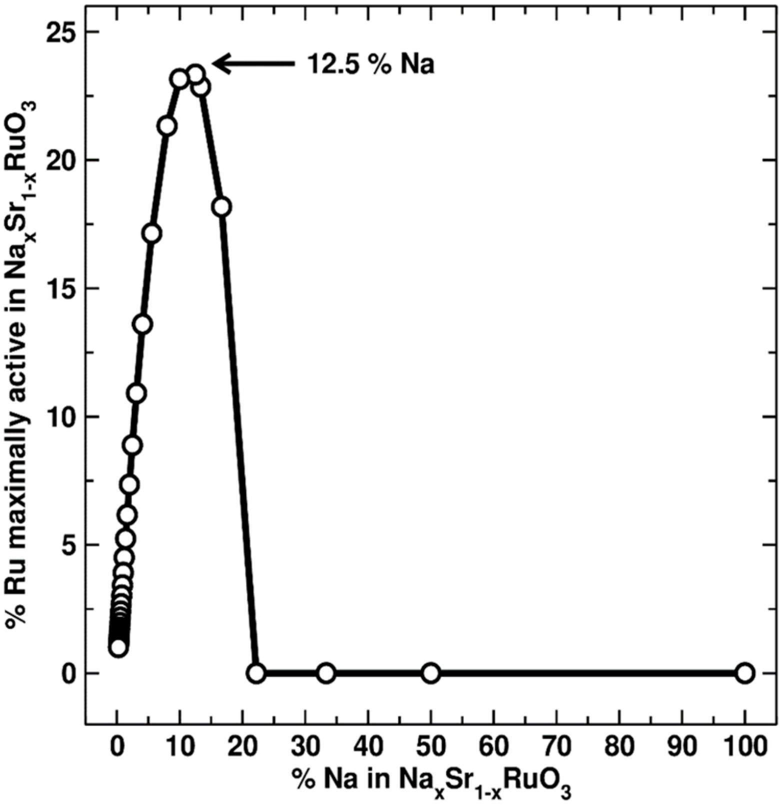

Supplementary Figure 13. Effect of the amount of Na doping in $Na_xSr_{1-x}RuO_3$ on the amount of Ru sites enhanced by the presence of Na.

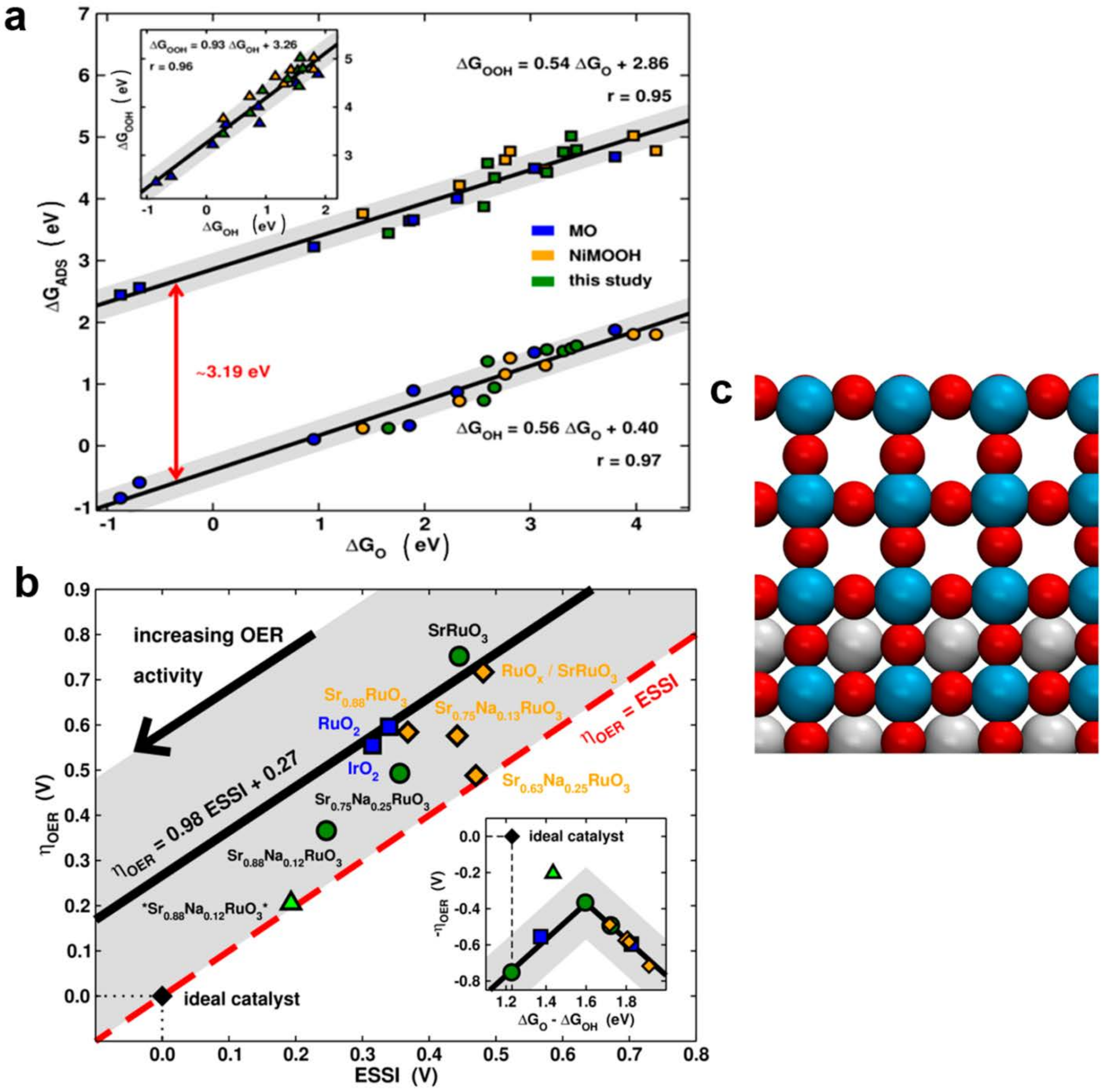

Supplementary Figure 14. Scaling relations, ESSI and side view of a RuO_3 overlayer. **a** Scaling relations between *O, *OH, and *OOH. We provide correlation coefficients, least-squares linear fits and a grey area of ± 0.2 eV around those. For comparison, data for MO (with M = Ti to Cu) and NiMOOH (M = Cr to Zn) are provided.[18] **b** ESSI analysis for the OER on the compounds under study. The correlation between ESSI and η_{OER} was taken from ref. [20]. Inset: conventional volcano plot (same data as in Fig. 2c in the main text) provided for comparison. *$Sr_{0.88}Na_{0.12}RuO_3$* is the optimized counterpart of $Sr_{0.88}Na_{0.12}RuO_3$. **c** Side view of a RuO_3 overlayer on top of $SrRuO_3$ (denoted $RuO_x/SrRuO_3$) formed upon Sr/Na leaching under OER conditions. White: Sr, red: O, blue: Ru.

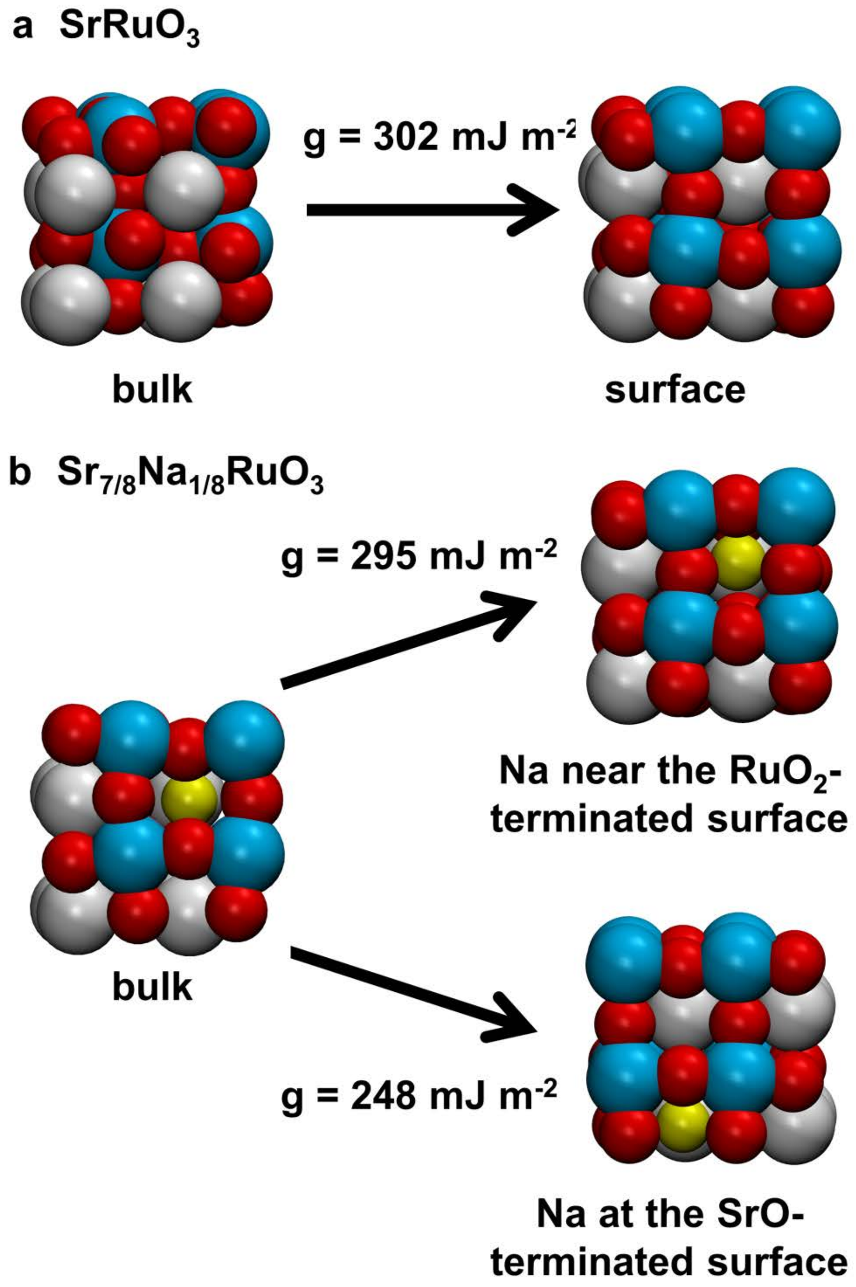

Supplementary Figure 15. Surface energies for the perovskites under study. **a** Pristine $SrRuO_3$. b) Na-doped $SrRuO_3$. In the latter, the formation energies of the RuO_2-terminated (top) and SrO-terminated (bottom) surfaces are provided. The color code in this figure is the same as in Supplementary Figure 5.

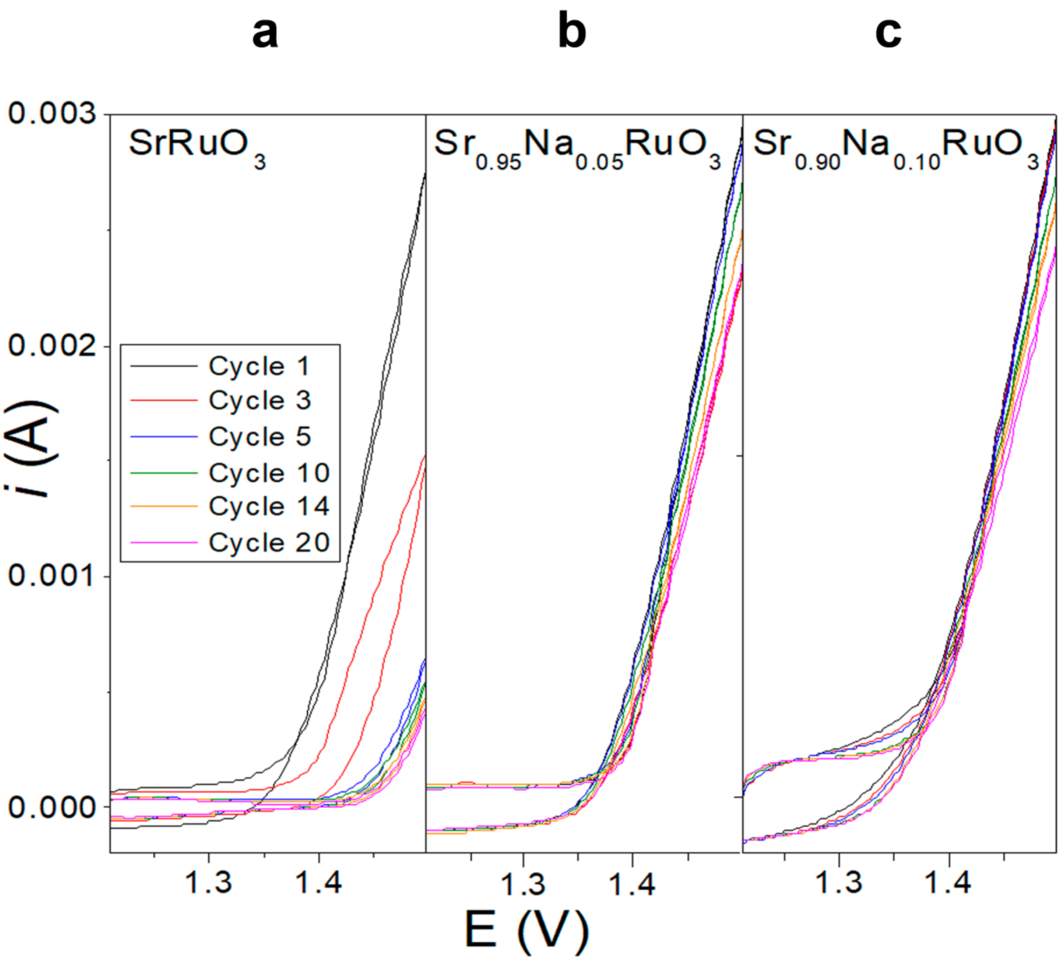

Supplementary Figure 16. 20 cyclic voltammograms between 1.2-1.5 V in O_2 saturated 0.1 M $HClO_4$ at 10 mVs^{-1} and 1600 rpm. **a** $SrRuO_3$, **b** $Sr_{0.95}Na_{0.05}RuO_3$ and **c** $Sr_{0.90}Na_{0.10}RuO_3$.

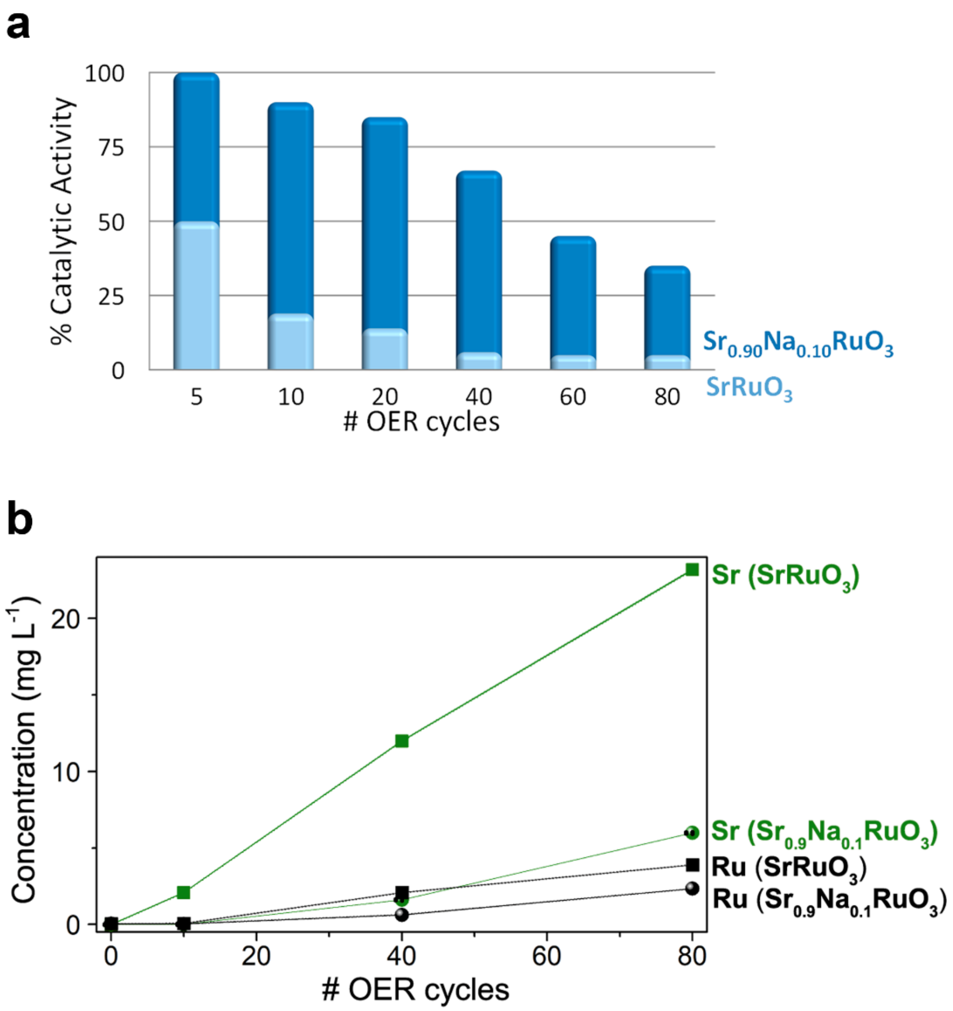

Supplementary Figure 17. Catalysts stability and ICP-OES analysis of the electrolyte. **a** Evolution of the catalytic activity during 80 OER cycles from 1.1 V to 1.5 V at 10mV/s of $SrRuO_3$ and $Sr_{0.90}Na_{0.10}RuO_3$. **b** ICP-OES analysis of the electrolyte before reaction and after 10, 40 and 80 OER reaction cycles on $SrRuO_3$ and $Sr_{0.90}Na_{0.10}RuO_3$ samples, showing the degree of dissolution of Sr and Ru on the electrolyte for both samples.

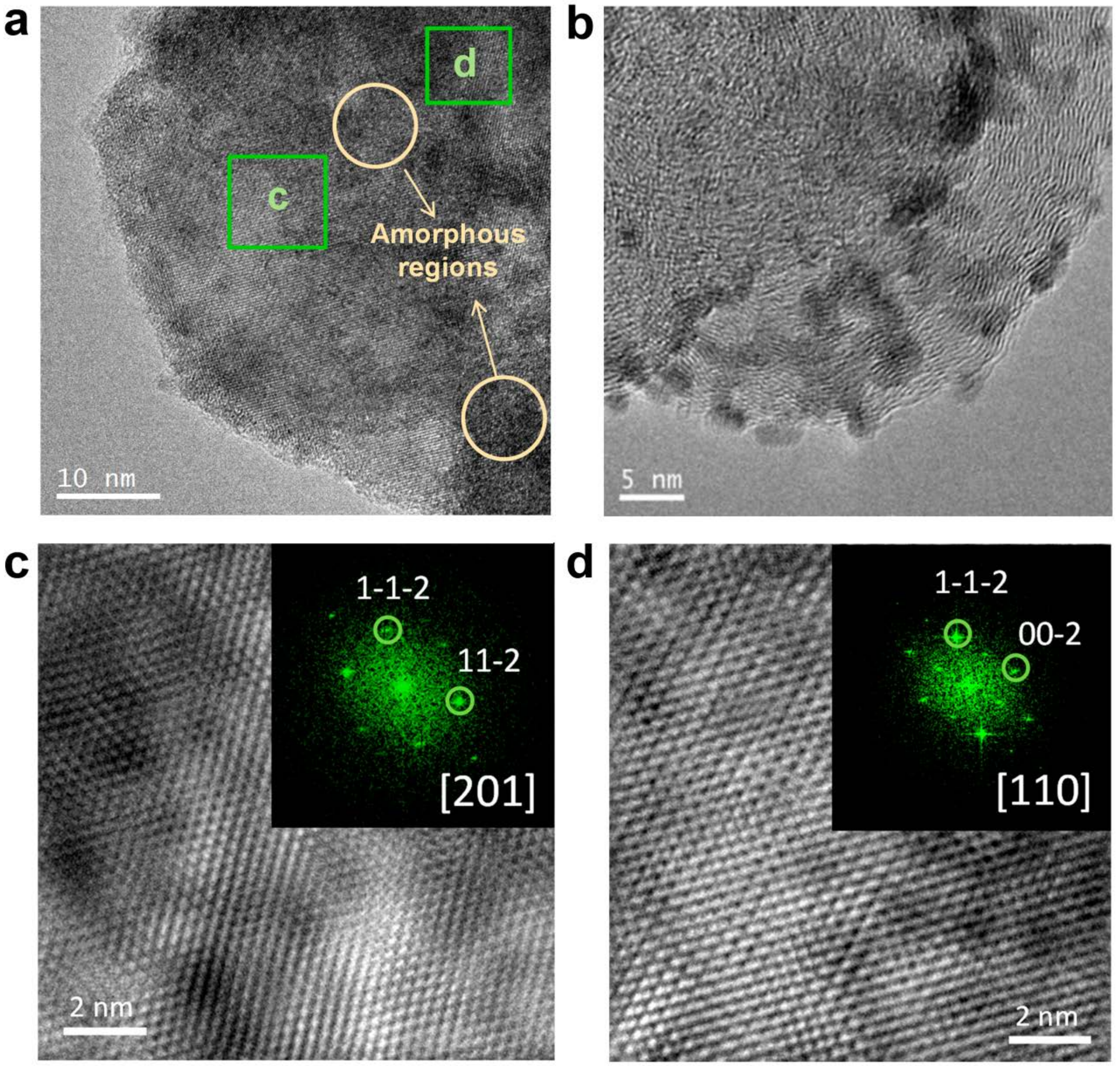

Supplementary Figure 18. TEM and HRTEM/DDP images of $SrRuO_3$ recovered after 20 cycles in the OER. **a** Representative TEM image. **b** TEM image showing individual RuO_x particles. **c** and **d** HRTEM and DDP images of the squared nano-domains from (a) with different orientations. Both zone axes correspond to perovskite structure.

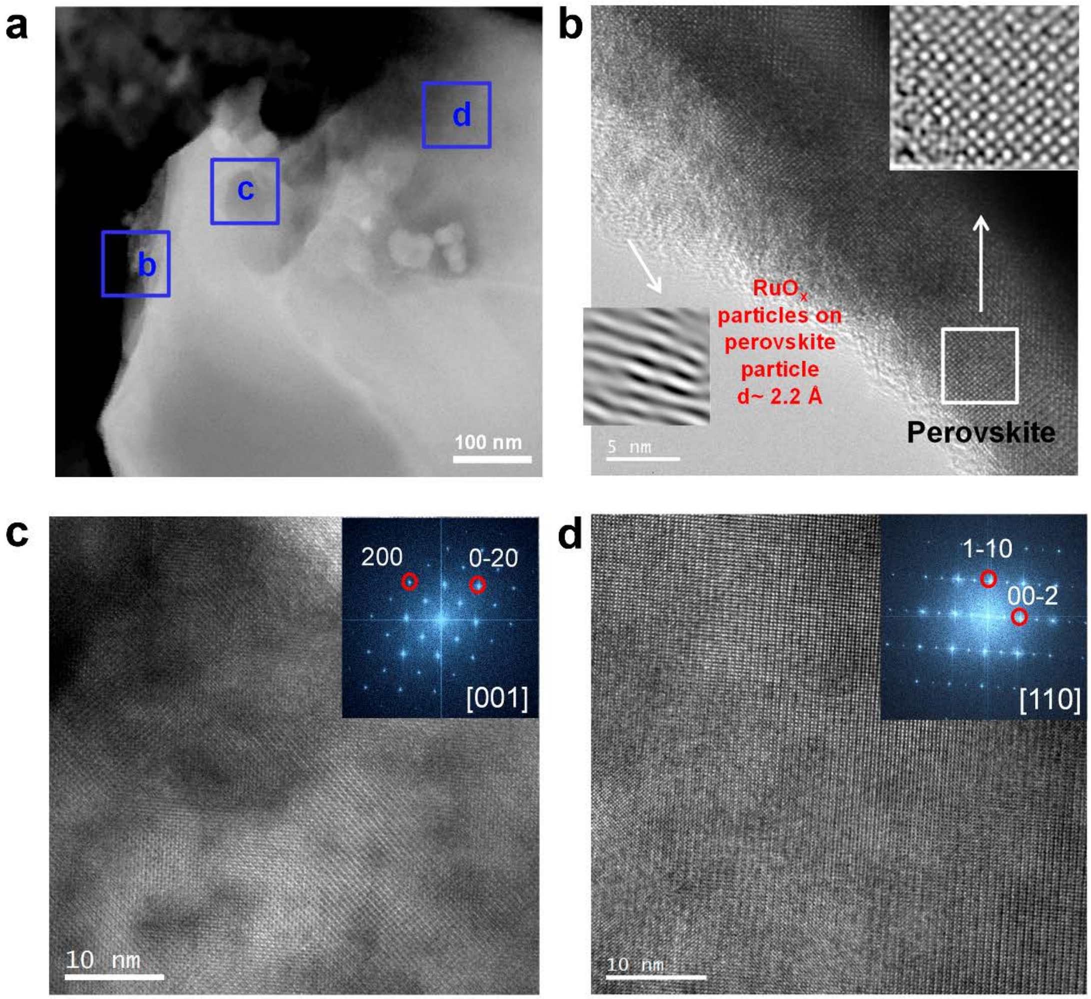

Supplementary Figure 19. STEM, TEM and HRTEM/DDP images of $Sr_{0.90}Na_{0.10}RuO_3$ recovered after 20 OER cycles. **a** Representative STEM image highlighting b, c and d regions. **b** HRTEM image and filtered images of region b in (a). The filtered images show an area with the incipient formation of RuO_x and an area preserving perovskite structure. **c** and **d** HRTEM/DDP for the regions c and d in (a) showing two zone axes of the perovskite structure. The crystallinity of the perovskites is demonstrated, note the different orientations.

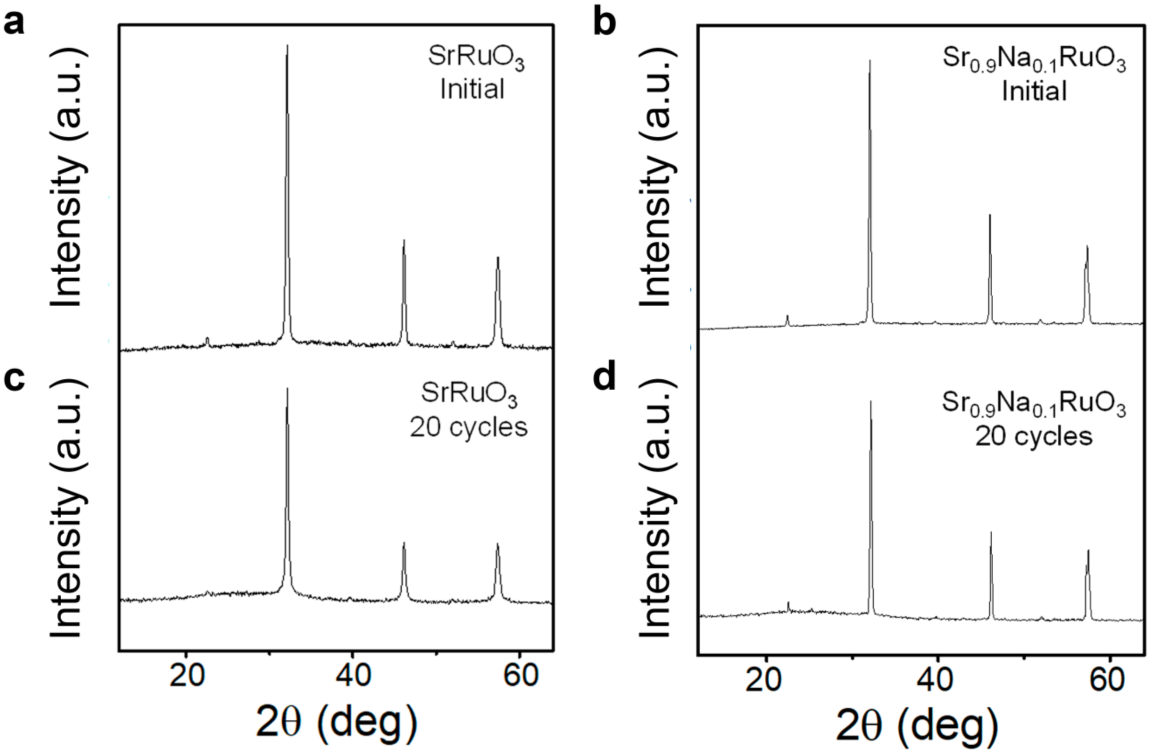

Supplementary Figure 20. X-ray Diffraction Patterns (XRD). **a** XRD of initial $SrRuO_3$. **b** XRD of initial $Sr_{0.90}Na_{0.10}RuO_3$. **c** XRD of $SrRuO_3$ after 20 cycles of OER reaction. **d** XRD of $Sr_{0.90}Na_{0.10}RuO_3$ after 20 cycles of OER reaction.

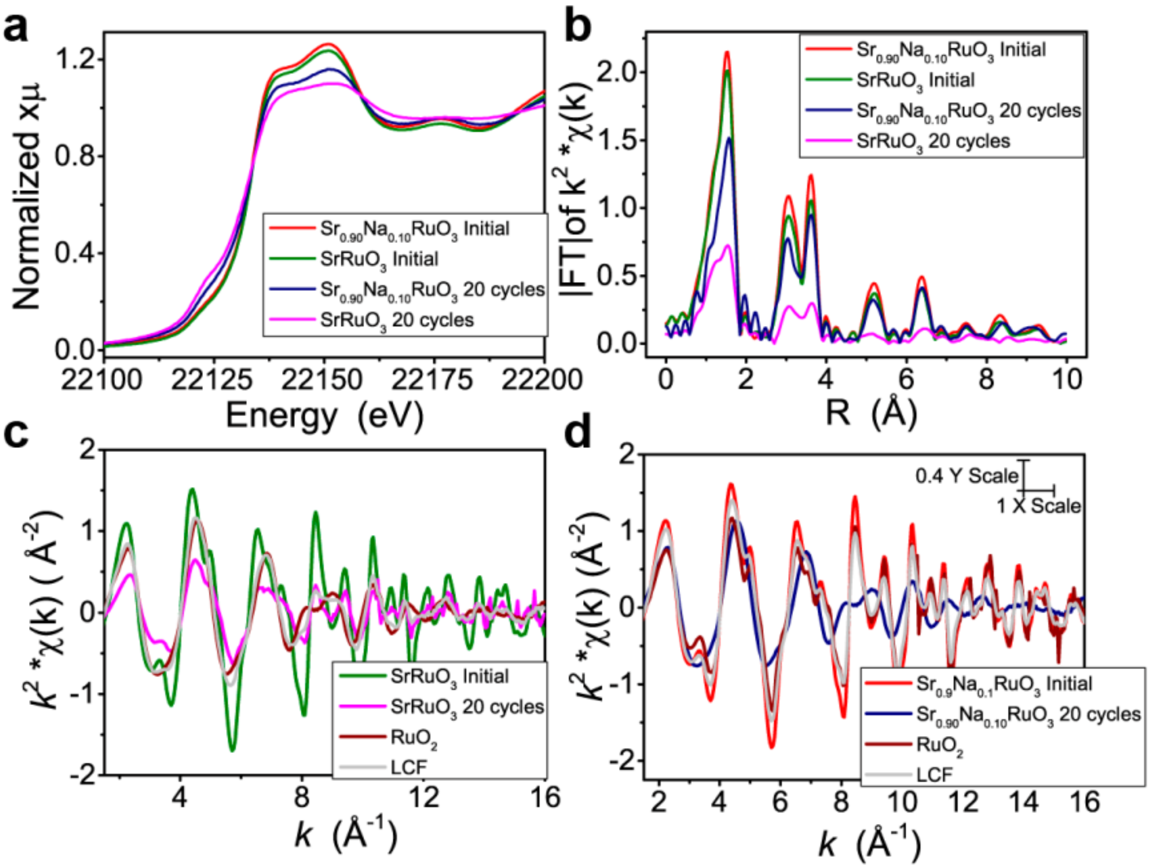

Supplementary Figure 21. X-ray Absorption Spectroscopy. **a** Ru K-edge XANES spectra of $SrRuO_3$ and $Sr_{0.90}Na_{0.10}RuO_3$ before and after 20 cycles of OER reaction. **b** Fourier Transform from Ru K-edge EXAFS signal before and after 20 cycles. Lineal combination (LCF) of the EXAFS signals for **c** $SrRuO_3$ before and after 20 cycles of OER reaction and **d** $Sr_{0.90}Na_{0.10}RuO_3$ before and after 20 cycles of OER reaction.

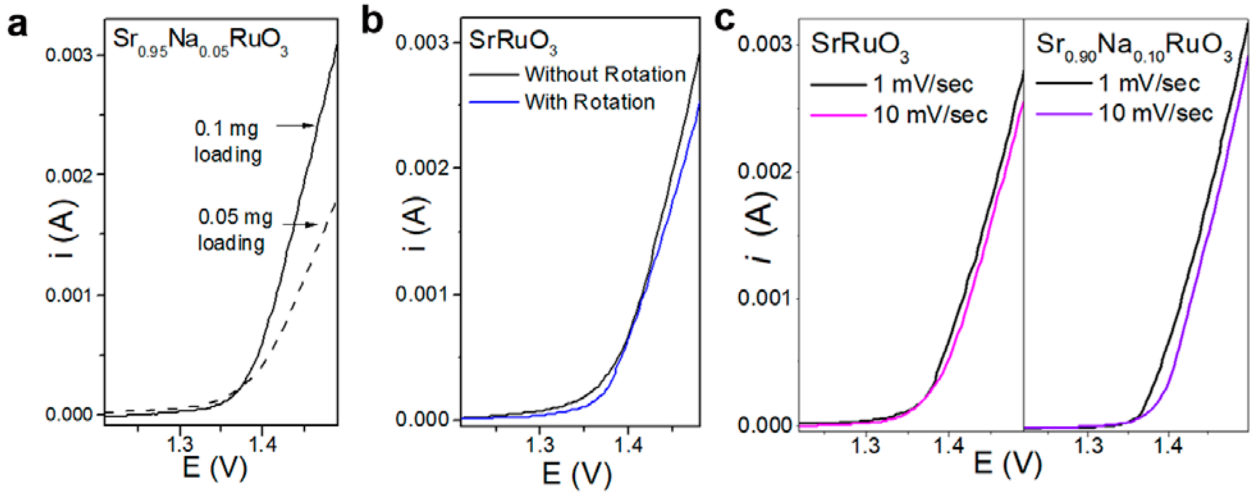

Supplementary Figure 22. Assessment of mass transfer effect. **a** OER with 0.1 and 0.05 mg $Sr_{0.95}Na_{0.05}RuO_3$ catalyst loading. **b** OER measurements for $SrRuO_3$ with 1600 rpm rotation rate and without rotation. **c** OER at 1 and 10 mVs^{-1} scan rates for $SrRuO_3$ (left panel) and $Sr_{0.90}Na_{0.10}RuO_3$ (right panel).

Supplementary Table 1. Atomic parameters after the refinement of the crystallographic structure of $Sr_{0.85}Na_{0.15}RuO_3$ from high resolution PND data at T = 295 K. Space group P*bnm*. Lattice parameters: a = 5.57206(6) Å, b = 5.53285(6) Å, c = 7.84947(8) Å and V = 241.994(4) $Å^3$. Discrepancy factors: R_{bragg} = 3.71, R_p = 4.03 %, R_{wp}= 5.10 %, χ^2 = 2.05.

**Atom**	**Site**	**x**	**y**	**z**	f_{occ}	**B(Å^2)**
Sr/Na	4c	-0.0009(3)	0.0163(3)	1/4	0.924(2)/0.076(2)	0.32(2)
Ru	4b	1/2	0	0	1.0	0.32(2)
O1	8d	0.7234(2)	0.2771(2)	0.02688(15)	1.000(1)	0.55(2)
O2	4c	0.0536(3)	0.4973(4)	1/4	0.998(2)	0.55(2)

Supplementary Table 2. Atomic distances (Å) for RuO_6 octahedra in $Sr_{0.85}Na_{0.15}RuO_3$.

**RuO_6 Octahedra**	
Ru – O1 (x2)	1.9860(11)
Ru – O1 (x2)	1.9853(11)
Ru – O2 (x2)	1.9850(3)
<Ru – O>	1.9854(4)

Supplementary Table 3. Specific surface area (BET), electrochemical surface area (ECSA), volume/area diameter ($d_{v/a}$) and particle size distribution (As) for $Sr_{0.85}Na_{0.15}RuO_3$

**Sample**	**BET / m^2g^{-1}**	**ECSA / cm^2**	**$d_{v/a}$ / µm**	**A_S / m^2g^{-1}**
$Sr_{0.85}Na_{0.15}RuO_3$	0.47(5)	0.23	2.8	0.3

Supplementary Table 4. Cell parameters, atomic positions, atomic occupancies, thermal parameters, selected interatomic distances and discrepancy factors for $Sr_{1-x}Na_xRuO_3$ refined from PND at 295 K in the *Pbnm* space group.

	**$SrRuO_3$**	**$Sr_{0.95}Na_{0.05}RuO_3$**	**$Sr_{0.90}Na_{0.10}RuO_3$**
a/Å	5.571(1)	5.5717(2)	5.57166(7)
b/Å	5.551(1)	5.5327(2)	5.53272(7)
c/Å	7.858(2)	7.8488(2)	7.8492(1)
V/Å^3	243.00(8)	241.95(1)	241.962(5)
Sr/Na 4*c* (*x y* ¼)			
x	0.0057(7)	-0.0005(9)	-0.0005(4)
y	0.004(1)	0.0179(7)	0.0173(3)
B_{iso}/Å^2	0.49(9)	0.35(4)	0.14(2)
Occ. Sr/Na	1.000/0.000	0.936(7)/0.064(7)	0.906(1)/0.094(1)
Ru 4*b* (½ 0 0)			
B_{iso}/Å^2	0.49(9)	0.35(4)	0.14(2)
O1 8*d* (*x y z*)			
x	0.7349(5)	0.7238(5)	0.7234(2)
y	0.2724(4)	0.2778(5)	0.2774(2)
z	0.0203(2)	0.0270(3)	0.0269(2)
B_{iso}/Å^2	0.97(2)	0.66(5)	0.44(2)
Occupancy O1*	1.009(5)	1.00(8)	1.000(2)
O2 4*c* (*x y* ¼)			
x	0.0601(4)	0.0536(8)	0.0549(3)
y	0.507(1)	0.498(1)	0.4973(4)
B_{iso}/Å^2	0.97(2)	0.66(5)	0.44(2)
Occupancy O2*	0.995(5)	0.996(8)	1.001(1)
RuO_6 Octahedra			
Ru –O1 (× 2)	2.006(2)	1.990(3)	1.988(1)
Ru –O1 (× 2)	1.950(2)	1.981(3)	1.984(1)
Ru –O2 (× 2)	1.9933(6)	1.9848(7)	1.9860(3)
<Ru –O>	1.9831(9)	1.9855(9)	1.9858(4)
R_{Bragg}	4.67	4.25	4.29
χ^2	1.84	1.29	2.19
R_p/%	2.40	3.66	4.18
R_{wp}/%	3.20	4.63	5.27

Supplementary Table 5. Sr/Na ratios determined from ICP and PND

Nominal Content	ICP Analysis	PND Occ. Sr/Na*
$Sr_{0.95}/Na_{0.05}$	$Sr_{0.949(9)}/Na_{0.051(2)}$	$Sr_{0.936(7)}/Na_{0.064(7)}$
$Sr_{0.90}/Na_{0.10}$	$Sr_{0.93(4)}/Na_{0.06(2)}$	$Sr_{0.906(1)}/Na_{0.094(1)}$

* See Supplementary Table 4

Supplementary Table 6. Formation energies of oxygen vacancies for the perovskites under study.

**$\Delta E_{O,vac}$ in eV**	**$Sr_8Ru_8O_{24}$ → $Sr_8Ru_8O_{23}$**	**$Sr_7Na_1Ru_8O_{24}$ → $Sr_7Na_1Ru_8O_{23}$**
Bulk	2.43	1.94
SrO-terminated surface	2.19	2.11
RuO_2-terminated surface	1.50	1.75

Supplementary Table 7. Specific surface area (BET), electrochemical surface area (ECSA), volume/area diameter ($d_{v/a}$) and particle size distribution (As) for $Sr_{1-x}Na_xRuO_3$.

Sample	BET / m^2g^{-1}	ECSA / cm^2	$d_{v/a}$ / µm	A_S / m^2g^{-1}
$SrRuO_3$	6.47(3)	4.5	0.16	5.8
$Sr_{0.95}Na_{0.05}RuO_3$	0.36(2)	0.32	1.10	0.9
$Sr_{0.90}Na_{0.10}RuO_3$	0.39(4)	0.48	2.8	0.3

Supplementary Table 8. Binding energies (eV) of $SrRuO_3$ and $Sr_{0.90}Na_{0.10}RuO_3$ samples.

**Sample**	**Binding Energy / eV**		**Ru/Sr surface atomic ratio**
	**Sr3*d*$_{5/2}$**	**Ru3*p*$_{3/2}$**	
$SrRuO_3$ initial	132.1 (55)<br>134.1 (45)	464.0	1.0
$Sr_{0.90}Na_{0.10}RuO_3$ initial	132.2 (70)<br>133.7 (30)	463.8	0.9
$SrRuO_3$, 20 cycles	132.8 (52)<br>134.3 (48)	464.8	2.7
$Sr_{0.90}Na_{0.10}RuO_3$, 20 cycles	132.5 (53)<br>134.6 (47)	464.6	1.8

*Values in parentheses show the relative atomic concentration of each species.

Supplementary Note 1: Characterization of fresh perovskites (composition and electron microscopy)

The chemical composition of the perovskites was analyzed by EDX and ICP-OES. The compositions obtained by EDX are: $Sr_{1.00(2)}Ru_{0.99(3)}O_y$ for $SrRuO_3$; $Sr_{0.97(5)}Na_{0.015(20)}Ru_{0.96(9)}O_y$ for x = 0.05; and $Sr_{0.99(2)}Na_{0.019(15)}Ru_{0.99(3)}O_y$ for x = 0.10. The Sr/Ru ratios obtained are in the range between 0.99 and 1.12, in good agreement with the nominal value of 1. Although the evolution of the Sr/Na ratio is in agreement with the nominal values, the actual Na content estimated by EDX is lower than expected, probably due to the very low concentration of Na in the samples and Na evaporation under the electron beam. We have also analyzed the Sr/Na ratios by ICP-OES: $Sr_{0.949(9)}/Na_{0.051(2)}$ for x = 0.05 and $Sr_{0.93(4)}/Na_{0.06(2)}$ for x = 0.10. The surface atomic Ru/Sr ratio of the perovskites determined by XPS ranges between 0.95 and 1.0 for all samples.

The HRTEM images and the corresponding electron diffraction diagrams (SAED) for $SrRuO_3$ and $Sr_{0.90}Na_{0.10}RuO_3$ indicate the formation of stoichiometric perovskites without vacancies (Supplementary Figure 4). The formation of vacancies, when they are ordered, would result in the observation of additional reflections (spots or diffuse scattering), corresponding to the formation of super-cell structures or short-range order, respectively. The SAED analyses fail to show diffractions spots other than those for the stoichiometric perovskite structure.

Supplementary Note 2. Results for $Sr_{0.85}Na_{0.15}RuO_3$

We tried to synthesize $Sr_{0.85}Na_{0.15}RuO_3$, but we observed that the amount of Na actually incorporated in the sample is significantly smaller than x = 0.15. This is probably because of the high temperature used for the synthesis of this sample (1050ºC vs 1000 ºC used for the synthesis of x = 0.10). Nevertheless, we include this sample in our work because despite the lower content of Na in this sample, it also shows that Na incorporation increases the stability of $SrRuO_3$ during the OER in acid.

The chemical composition by NPD is ICP-OES is $Sr_{0.91(4)}/Na_{0.06(2)}$, *i.e.*, the actual x = 0.05, lower than the intended (nominal) value of x = 0.15.

Supplementary Note 3. Oxygen vacancies formation energies

In order to assess feasibility of oxygen vacancies on Na-doped $SrRuO_3$, we performed theoretical calculations of the energy necessary to create oxygen vacancies at the surface and the bulk of $SrRuO_3$ and $Sr_{0.875}Na_{0.125}RuO_3$ on bulk and slab cells containing 8 formula units. The large values in Supplementary Table 6 confirm the unlikely formation of oxygen vacancies in our samples. Note that these formation energies are in good agreement with similar experimental and computational values reported in the literature for perovskites that do not exhibit oxygen vacancies.[9] This supports the idea that Na incorporation is not compensated by generating vacancies but by a partial oxidation of Ru atoms in Na-doped perovskites. Oxygen vacancies are typically formed more easily at the surface compared to the bulk, and this is verified on undoped $SrRuO_3$. However, Na doping largely stabilizes SrO-terminated surfaces, making them less prone to releasing oxygen compared to the bulk.

In Supplementary Table 6 one can also observe that while the bulk of undoped $SrRuO_3$ has more positive oxygen-vacancy formation energies than the doped one, the SrO-terminated surfaces exhibit comparable stability and, interestingly, the RuO_2-terminated surface is more stable against oxygen release upon Na doping. The configurations of the oxygen vacancies are show in Supplementary Figure 5.

Supplementary Note 4. X-ray Absorption Spectroscopy (XAS) of fresh perovskites.

Soft and Hard X-rays have been used to carry out XAS experiments in different energy ranges. Supplementary Figure 6a shows Sr-L3, Sr-L2 and Na K-edges. Data from Na K-edge spectra confirm the presence of Na, but an absolute quantification of the sodium content was not possible. The Na-edge normalization became difficult to perform due to the low signal level observed and because the small k-range measured as the signal overlaps with the Sr K-edge background signal (L3- and L2-edges). Besides, these signals have been measured in fluorescence mode, so artefacts coming from self-absorption effects can affect the spectra, especially on the Sr element.

Ru K-edge data were collected in a range up to 18 $Å^{-1}$. In Supplementary Figure 6b, the EXAFS data has been Fourier transformed to obtain the radial distribution function (RDF) (phase non-corrected). The first maximum is related to the first coordination shell and the second one to the scattering of the atoms located at greater distances as Ru-Ru or Ru-Sr or multiple scattering paths involving more than two atoms. As observed in Supplementary Figure 6b, the FT-transformed EXAFS signal from the doped samples are more intense than the signal for $SrRuO_3$. Although this behaviour could be related to the presence of oxygen vacancies, our PND and HRTEM results clearly demonstrate the lack of vacancies. Therefore, the lower intensity of the signals for $SrRuO_3$ account to a higher level of distortion of RuO_6 octahedra in $SrRuO_3$. In general, for the doped samples, as the Ru-O-Ru chain presents higher linearity by symmetry, the intensity in this EXAFS-signal range would be increased and the low dopant levels make no significant effect on the single scattering contribution at higher shells.[1]

The XANES Ru K-edge experimental spectra (Supplementary Figure 7a) show excellent agreement with the simulated spectra obtained from the structural model determined by PND, confirming that the changes observed in the XANES are due to the incorporation of Na into the perovskite structure and the reduction of octahedra distortions. Simulated spectra (Supplementary Figure 7b) confirm a clear difference on the relative intensities of the A and B features between the doped and undoped samples. This difference is associated with a different distribution of the charges. The 5*px(y)* level presents more electronic density in the Na-doped samples and the 5*pz* level presents more electronic density in the undoped sample. The relative change in intensities seems to be proportional to the Na amount measured by PND ratio (Supplementary Figure 7c).

We have also analyzed the pre-edge region of the Ru K-edge (Inset Supplementary Figure 7a), since it could be associated to Na^{+} insertion. The slight differences observed in the experimental pre-edge signal intensities match the simulation results. The simulations show that this feature can be associated to a quadrupole electron transition from 1s to 4d Ru orbital. The small enhancement of the experimental and simulated doped sample signal intensities are correlated with a higher probability for the electrons to be promoted to the 4d Ru orbital. This difference in the electronic distribution around Ru can be explained with a slightly positive charge environment caused by insertion of Na dopants. Even if it was not possible to observe a shift of the oxidation state in this edge, this result is in agreement with the evolution of oxidation states evidenced by the shift of the Ru $M_{2,3}$-edge and PND.

Supplementary Note 5. Surface Area Calculation

The current densities (j) were calculated by normalizing the obtained current to the geometric area of the electrode (j in $mAcm^{-2}_{geo}$). The intrinsic OER activity (i_s) was determined normalizing the obtained currents by the surface area. In the main text we used A_S for the normalization. There are different ways to calculate this area:

a) BET method

The results obtained for the specific surface areas from BET are detailed in Suplementary Table 7 and the normalization of the activity in Supplementary Figure 8.

b) Particle size distribution by TEM (A_S)

Another way to determine the surface area of oxides is using the particle size distribution and obtaining the mass-specific surface area (A_S).[2] We calculated the diameter of the particles by TEM (approximating to a spherical geometry) and then the volume/area diameter ($d_{v/a}$) as $d_{v/a}= \Sigma d^3/\Sigma d^2$. A_S was determined using the following formula:

$$A_s = \sum \pi d^2 \Big/ \sum \tfrac{1}{6} \rho \pi d^3 = 6 \sum d^2 \Big/ \rho \sum d^3 = 6 \big/ \rho d_{v/a} \qquad (1)$$

where d is the average diameter of the particles calculated by TEM (Supplementary Table 7) and ρ is the oxide bulk density calculated for $Sr_{1-x}Na_xRuO_3$ (P*bnm*) following the formula:

$$\rho = \frac{Mw \times Z}{N_A \times V_{f.u}} \qquad (2)$$

Where Mw is the molecular weight, Z is the number of formula units per cell, N_A is Avogadro's number, and $V_{f.u.}$ is the volume of the formula unit. The values obtained for A_S are shown in Supplemantary Table 7 and the normalization of the activity in Supplementary Figure 8.

c) Electrochemical Active Surface Area (ECSA)

The electrochemical active surface areas (ECSA) were calculated by the double-layer capacitance of the surface of each compound. We performed cyclic voltammograms around the open circuit potential in Argon (from 0.6 and 0.9 V), obtained curves in which the only proccesses occurring are suppossed to be due to the double-layer charging. The measurements are carried out at multiple scan rates (5, 10, 25, 50, 100, 200 and 400 mV/s) and the double-layer charging current (i_c) is equal to the product of the scan rate (ν) and the double-layer capacitance

(C_{dl}) following the equation: $i_c = \nu C_{dl}$. The plot of i_c *vs.* ν at a constant potential gives the capacitance, C_{dl}¸ as the slope; so that the ECSA is: ECSA= C_{dl}/ C_S, where C_S is the specific capacitance of an atomically flat planar surface of the compounds per unit area under the same electrolyte conditions. We used C_S= 0.06 mF/cm^2, following several references for similar oxides measured on 0.1M $HClO_4$.[3-5] The ECSA values obtained are shown in Supplementary Table 7 and the normalization of the specific catalytic activity by ECSA is shown in Supplementary Figure 8, to compare the obtained activities with the ones obtained from A_S and BET normalizations.

Supplementary Note 6. RRDE experiments

To evaluate whether the current recorded during the OER accounts to O_2 evolution we used a rotating ring disk electrode (RRDE) equipped with a Pt-ring. Two set of experiments were conducted. First, we performed a linear voltammograms keeping the Pt-ring at 1.1V *vs.* RHE. At such high positive potential, the oxygen reduction reaction (ORR) on Pt is negligible so the reducing current at the ring should account to the reduction of corrosion species (RuO_4^-) at the Pt ring. In a further experiment, we set the Pt ring at 0.4 V where the ORR takes place. Supplementary Figure 9 shows the results obtained for $Sr_{0.90}Na_{0.10}RuO_3$ as an example.

As observed, the current recorded by setting the Pt ring at 0.4 V is significantly higher than that recorded at 1.1 V, indicating that O_2 is produced during the OER. Note that the actual concentration of RuO_4^- is the same in both cases and independent of the Pt ring potential. In line with previous reports,[8] we determined that around 12% of the total current accounts to corrosion.

Supplementary Note 7. *In situ* Mass Spectrometry measurements

We further confirmed the formation of oxygen during the OER by *in situ* mass spectrometry (MS) experiments, by placing a MS probe in the vicinity of the working electrode during the OER. A quadrupole mass spectrometer (Pfeiffer Vacuum Prisma QME 200), with a range of 1-100 amu and a secondary electron multiplier / Faraday cup detector was used. The MS analysis chamber was pumped by a Pfeiffer Turbodrag pump TMU 071 P backed by a Pfeiffer diaphragm pump MVP 015-2, and was connected to the electrochemical cell by means of a 2 m long, 150 µm I.D. glass capillary. The capillary open end that is opposite to the analysis chamber was sealed with two layers of a porous PTFE membrane (Goodfellow FP301276, 0.085mm thickness, 0.02 µm pore size, 50% porosity), working as a gas/vapor probe. The probe was immersed in the electrolyte of the electrochemical cell. With this set-up, a stable pressure of $9.5 \cdot 10^{-6}$ mbar was kept in the MS analysis chamber during the experiment, and the analysis of the gases solved in the electrolyte (e.g., oxygen after its evolution) is possible. The glass atmosphere-controlled electrochemical cell contained 100 mL of a 0.1 M $HClO_4$ aqueous solution as electrolyte, with a silver/silver chloride reference electrode and a graphite counter-electrode. The working electrode was prepared adding the electrocatalyst ink to both faces of a 1 mm thin graphite wafer, with a total surface of 500 mm^2. The total catalysts load was of ~5 mg, equally distributed on both sides. The electrolyte gas outgassed by bubbling argon under stirring, until the signals in the MS of Ar (m/z=40), O_2 (m/z=32) and N_2 (m/z=28) were stable (typically, 30 min). Then Ar gas flow was maintained over the electrolyte, as well as the stirring, and the electrochemical measurements were carried out at room temperature using an Autolab PGstat 100N potenciostat. I-V curves (linear single scan, LSV) were obtained at a scan rate of 0.1 mV/s in the range 1.3-1.5 V for the $Sr_{0.9}Na_{0.1}RuO_3$ perovskite and 1.3-1.65 V for IrO_2 reference electrocatalyst.

Supplementary Figure 10 shows the O_2/N_2 MS signals ratio *vs.* the time of the OER experiment for $Sr_{0.9}Na_{0.1}RuO_3$. The background ratio (*ca.* 0.35) corresponds to the typical O_2/N_2 ratio of the residual gases in the MS analysis chamber under the analysis conditions. The enhancement of this ratio clearly demonstrates an increment of the O_2 production during the OER. The corresponding I-E curve is depicted below. As observed in Supplementary Figure 10a, the production of O_2 follows the pattern of the current recorded during the OER. Note that the slight delay in O_2 production could be intrinsic to our experimental setup. Note also that this

experiment aims to prove O_2 evolution during OER, but is not intended to give accurate information about the overpotentials at which the OER proceed nor to quantify O_2 production.

In order to assess that the observed O_2 production is not due to the entrance of air in the cell during the experiment, we have normalized the O_2 signal to the signal for N_2 (m/z=28) and, for the sake of comparison, we have monitored O_2 evolution with IrO_2 using the same experimental conditions (Supplementary Figure 10b).

Supplementary Note 8. Effect of Na doping on the electronic structure of $SrRuO_3$

Let us consider the case of $SrRuO_3$ and $Sr_{0.875}Na_{0.125}RuO_3$, namely a doped perovskite with 1/8 of the Sr sites substituted by Na. In the former compound, the oxidation state of Ru is +4, while it supposedly is +4.125 in the latter. A global oxidation state of +4.125 corresponds in reality to having 7/8 of all Ru sites in the +4 oxidation state and 1/8 of them in the +5 state.

Note that Rossmeisl et al.[30] have shown for transition metals (M), monoxides (MO, with M^{2+}), $LaMO_3$ (with M^{3+}) and $SrMO_3$ (with M^{4+}) that the adsorption energies of the OER intermediates (*O, *OH, *OOH) decrease linearly as a function of the oxidation state of the M sites. Thus, it is expectable that the adsorption energies of the OER intermediates on Ru^{4+} sites be stronger (more negative) than those of Ru^{5+} sites. This trend essentially reflects the fact that cationic sites in a lattice become less willing to create bonds with highly electrophilic adsorbates as their oxidation state within such lattice increases.

When oxidizing Ru^{4+} to Ru^{5+} sites, the metal d-band empties from d^4 to d^3, which should result in a sizable displacement of Ru's d-band center towards more positive values. Shao-Horn and coworkers[31] have also pointed out that because of the hybridization between the O p-band and the Ru d-band, the d-band shift should be accompanied by a shift in the O p-band, and the latter should be better described by DFT+U (i.e. the methodology used in their work and in here).

All these ideas are summarized in Supplementary Figure 11, where we show that: 1) Na doping displaces positively the O p-band and Ru d-band centers in $SrRuO_3$, 2) the changes depend gradually on the amount of Na doped, and 3) the band center shifts are proportional to the weakening of the adsorption energies of *O and *OH, which are used as descriptors in the volcano plot in Figure 2c in the main text of the article.

Supplementary Note 9. Combinatorial model to estimate the optimal % Na doping

We have made a combinatorial analysis to show that the optimal configuration is indeed located at Na contents of 12.5%. The model is based on the fact that DFT shows that 1 Na ion adjacent to a Ru site takes it to the top of the OER volcano, but two Na ions push it further away ($Sr_{0.875}Na_{0.125}RuO_3$, $Sr_{0.75}Na_{0.25}RuO_3$ in Figure 2c in the main text of the article). Therefore, for a given % Na one can determine the % Ru which will likely be enhanced. We illustrate the approach in Supplementary Figure 12 for a 4×4 surface unit cell of $SrRuO_3$, where we show the non-equivalent configurations (taking into account that the cells are periodic) for 2 Na ions distributed among the 16 available sites, so that the % Na = (2/16)×100 = 12.5 %. There are 15 different configurations and seven of them, marked with (+) in Supplementary Figure 12, have Na maximally dispersed, so that no Ru atom is adjacent to more than 1 Na ion. In this context, (7/15) ×100 = 46.7% is the probability of finding two nonconsecutive Na ions for such total % Na. If (i) 46.7% of the Na atoms are nonconsecutive, (ii) the Na content is 12.5%, and (iii) each Na ion is adjacent to four Ru centers, the maximal amount of Ru centers predicted to be enhanced by Na doping is 12.5% × 46.7% × 4 = 23.33%.

Repeating the analysis on other cubic and rectangular cells (1×1, 2×2, 3×3… up to 28×28, and 2×3, 3×4, 3×5, 4×5), one obtains a plot correlating % Na and % Ru maximally active, as shown in Supplementary Figure 12.

Clearly, Supplementary Figure 13 shows that there needs to be a compromise in Na doping, as both high and low Na content reduce the probability of finding Ru sites with 1 adjacent Na only. The top of the curve is found for 12.5 % Na, as initially predicted by the volcano plot in Figure 2c of the main text, and it is observed that % Na in the range of 8-13.33% show similarly high amounts of active Ru sites, larger than 20%.

Supplementary Note 10. Surface energies and dissolution potentials

The surface energy (γ) of a material is typically calculated with respect to the energy of its bulk on a per formula unit (f. u.) basis and normalizing by the surface area, as shown in Equation 3.[10] As a formula unit of a given compound is usually more stable at the bulk of the material than at the surface, surface energies are positive quantities. One can rationalize the fact that surface energies be positive by considering that surfaces form by cutting bonds in the bulk, which costs energy (this is the basis of the bond-cutting model for surfaces).[11]

If surface energies are positive quantities measured with respect to the bulk, then the values for stable surfaces should be as close to zero as possible.

The surface energies of the ABO_3 compounds are defined as follows:

$$\gamma = \frac{E_{surf}^{f.u.} - E_{bulk}^{f.u.}}{2A_{surf}^{f.u.}} \tag{3}$$

Where the denominator is the surface area of a formula unit and the numerator is the difference between the total energies per formula unit of bulk ABO_3 and a slab of ABO_3 in which both an AO-terminated and a BO_2-terminated surfaces are present (hence the division by 2). In Supplementary Figure 15 we show the formation energies per formula unit for bulk and slabs with the formula $SrRuO_3$ and $Sr_{7/8}Na_{1/8}RuO_3$. We observe that Na lowers the surface energy of the slabs, especially in the SrO-terminated surfaces. However, note that in order to preserve the stoichiometry of the perovskite and because of the use of periodic boundary conditions, both surfaces are simultaneously formed in the slabs, so the formation energies of both surfaces are always present in our assessments.[12,13]

We have also calculated the potentials for Sr and Na dissolution from Na-doped $SrRuO_3$ and for Sr dissolution from $SrRuO_3$, combining DFT values and experimental data.[14] We note that the predicted formation energies of oxides with respect to the constituent elements are generally wrong at the GGA level, while they agree with experiments when they are calculated with respect to other oxides.[15] In other words, the DFT formation energy of $SrRuO_3$ with respect to Sr, Ru and O_2 is likely inaccurate, whereas that from SrO and RuO_2 is likely close to experiments. As discussed elsewhere,[15] that is probably due to error cancellation. Thus, we calculated the potentials below using DFT energies for SrO and Na_2O. The surface reactions considered are:

$$SrRuO_3 \rightarrow Sr_{0.5}RuO_3 + 0.5Sr^{2+} + 1e^{-} \tag{4}$$

$$Sr_{0.875}Na_{0.125}RuO_3 \rightarrow Sr_{0.4375}Na_{0.0625}RuO_3 + 0.4375Sr^{2+} + 0.0625Na^{+} + 0.9375e^{-} \quad (5)$$

$$Sr_{0.875}Na_{0.125}RuO_3 \rightarrow Sr_{0.5}RuO_3 + 0.375Sr^{2+} + 0.125Na^{+} + 0.875e^{-} \quad (6)$$

Equation (4) describes the dissolution of Sr^{2+} from pristine $SrRuO_3$, whereas (5)-(6) describe the progressive dissolution of Sr and Na for $Sr_{0.875}Na_{0.125}RuO_3$. We find that U_4 = 1.66 V, U_5 = 1.73 V, and U_6 = 1.76 V. The differences in the standard potentials of Equations (4)-(6) are, therefore, $U_5 - U_4 = 73$ mV and $U_6 - U_4 = 98$ mV, which means that (i) Sr dissolution is thermodynamically hindered upon Na doping and (ii) Na dissolution should, in principle, take place after Sr dissolution, as the dissolution potential for Na is more positive than that of Sr.
Besides, we have considered the following dissolution reaction:

$$Sr_{8-x}Na_xRu_8O_{24} + 2\left(H^{+} + e^{-}\right) \rightarrow Sr_{8-x}Na_xRu_8O_{23} + H_2O \quad (7)$$

Approximating the free energies of the solids to their total DFT energies and making use of the computational hydrogen electrode,[16] the energetics of this reaction is given as:

$$\Delta G_{diss,onset} = \left(E_{Sr_{8-x}Na_xRu_8O_{23}} + G_{H_2O} - E_{Sr_{8-x}Na_xRu_8O_{24}} - G_{H_2}\right) + 0.118\,pH \quad (8)$$

At pH = 1 (0.1 M $HClO_4$) and using a Na doping of 12.5% (1 Na atom per 7 Sr atoms), the stability of the SrO-terminated surface is comparable to that of undoped $SrRuO_3$: they differ only by 0.08 eV. However, the RuO_2-terminated surface is stabilized in presence of Na by 0.25 eV. In terms of the electrode potential, the latter difference is equivalent to 130 mV. Now, it is clear from the equation above that larger pH values prevent dissolution.
On the other hand, the full dissolution of the perovskite surfaces leads to the formation of RuO_2 and dissolved A-site cations,[17] as described by the following reaction (for x = 0 and 0.125):

$$Sr_{1-X}Na_XRuO_3 + 2H^{+} + Xe^{-} \rightarrow RuO_2 + \left(1-X\right)Sr^{2+} + XNa^{+} + H_2O \quad (9)$$

Again, approximating the free energies of the solids to their total DFT energies and using the computational hydrogen electrode,[16] the energetics of this reaction can be written as:

$$\Delta G_{diss,full} = \Delta G_{solid} + 2(1-X)U_{diss}^{Sr} + XU_{diss}^{Na} + 0.118\,pH + 0.059\log\left([Sr^{2+}]^{1-X}[Na^{+}]^{X}\right) + XeU \quad (10)$$

where ΔG_{solid} is defined as:

$$\Delta G_{solid} = E_{RuO_2} + \left(1-X\right)E_{Sr} + XE_{Na} + G_{H_2O} - E_{Sr_{1-X}Na_XRuO_3} - G_{H_2} \quad (11)$$

As our experiments were made in 0.1 M $HClO_4$ (pH = 1), the maximal final concentration of Sr^{2+} is 0.05 M when $SrRuO_3$ is dissolved (0.046 M with a Na doping of 12.5%). In such case, the full dissolution of RuO_2-terminated surfaces of doped $SrRuO_3$ is less favorable by 0.08 eV with

respect to undoped $SrRuO_3$ at U_4 = 1.66 V (-0.40 eV vs -0.32 eV). At that potential, the stability against dissolution of SrO-terminated surfaces is enhanced by Na doping by 0.13 eV with respect to undoped $SrRuO_3$ (-0.40 eV vs -0.27 eV).

In summary, Na doping stabilizes $SrRuO_3$ because it makes Sr, Na and O dissolution (as Sr^{2+}, Na^+ and H_2O) more difficult at the initial stages of catalyst degradation. When the dissolution of the perovskite is general and a RuO_2 phase is generated, Na doping hinders the process by stabilizing both RuO_2- and SrO-terminated surfaces.

Supplementary Note 11. Scaling relations, volcano plots and ESSI

In Supplementary Figure 14 we show that the data calculated in this work fulfils the adsorption-energy scaling relations reported in the literature.[18] The expected slope for the adsorption-energy scaling relations of *OH vs *O and *OOH vs *O is 0.5, so that the slopes of 0.56 and 0.54 found here are in good agreement. The slope of the relation of *OOH vs *OH was found to be 0.93, also in good agreement with the expected value of 1. Furthermore, the offset of the latter scaling relation is expected to be 3.2 ± 0.2 eV[12] so that our value of 3.19-3.26 eV (depending on whether the slope is taken as 1 or 0.93, see Supplementary Figure 14a) is also fine. Therefore, we conclude that our data fits well in the state-of-the-art scaling relations, so that it is possible to say that the reaction energies of the electrochemical steps of the OER are well described by adsorption-energy-based descriptors. This enables the use of volcano-type plots for the analysis of OER activity trends. Furthermore, there is a correlation between reaction energies and reaction barriers, which in principle can be expressed by means of Brønsted-Evans-Polanyi (BEP) relations. Several years ago, Vojvodic et al.[19] analyzed the behaviour of BEP relations for transition metal oxides. Among their main findings is the fact that perovskite oxides follow such relations. This supports the assumption that favourable adsorption thermodynamics should correspond to favourable adsorption kinetics.

Note that the volcano plot in Figure 2c of the main text was built using the scaling relations in Supplementary Figure 14a. The scattering in the latter introduces errors of approximately ± 0.2 eV on the adsorption energy predictions (and ± 0.2 V on the (over)potentials), seen in Supplementary Figure 14a by the grey areas around the linear fits.

Schmickler et al.[21] have argued that the extensive use of Sabatier-type activity plots has led to some abuses in electrocatalysis, especially for the hydrogen evolution reaction (HER) and the oxygen reduction reaction (ORR). The most common abuse is the disregard of the actual surface state under reaction conditions, which is different for various materials under HER or ORR conditions. For instance, while Pt and Au are metallic under those conditions, more reactive metals such as W or Mo are oxidized. Widespread volcano plots, however, were built assuming all transition metals in their pure metal phases.[22] Note that the case for the OER is different, as it is widely known that at the large positive potentials at which it takes place, the materials are always oxidized.[12,23]

Another assumption of volcano plots is that the prefactors are (nearly) identical, which may or may not be the case when one compares different elements: for instance, for the oxygen reduction reaction it is known that the prefactors for Os and Pt differ by 5 orders of magnitude.[24] However, the prefactors are on the same order of magnitude for Pt, various Pt-Ru alloys and Pd. This is a somewhat intuitive result considering transition-state theory, in which the prefactor depends on temperature and the entropy difference between transition and initial states. At a fixed temperature, a high degree of chemical and geometrical similarity between all of the compared active sites makes it reasonable to assume that the prefactors are comparable.

In this vein, we would like to stress that the volcano plot in Figure 2c of the main text mostly contains data for Ru sites at (001) surfaces of undoped and Na-doped $SrRuO_3$. Therefore, we believe that in this particular case the volcano-type activity plot is applicable.

Alternatively, we provide in Supplementary Figure 14b an OER-activity analysis that does not depend on scaling relations but confirms the predictions in Figure 2c of the main text. The descriptor in Supplementary Figure 14b is the electrochemical-step symmetry index (ESSI) and is defined as:

$$ESSI = \left(\frac{1}{n} \sum_{i}^{n} \Delta G_i^* \right) - E^0 \tag{12}$$

where ΔG_i^* are the adsorption energies in the reaction pathway $H_2O \rightarrow {*OH} \rightarrow {*O} \rightarrow {*OOH} \rightarrow O_2$ which are larger than $E^0 = 1.23$ V. For instance, consider the case of $Sr_{0.875}Na_{0.125}RuO_3$, which has *O, *OH and *OOH adsorption energies of 3.16, 1.56 and 4.43 eV. The corresponding OER reaction energies are 1.56, 1.60, 1.27 and 0.49 eV, so that $ESSI = (1.56 + 1.60 + 1.27)/3 - 1.23 = 0.25$ V and $\eta_{OER} = 1.60 - 1.23 = 0.37$ V. According to scaling relations $\Delta\Delta G_{OH} \approx \Delta\Delta G_{OOH} \approx 0.5\Delta\Delta G_O$, so that the overpotential can be lowered from 0.37 to 0.21 V strengthening the adsorption energy of *OH, *OOH and *O by 0.16, 0.16 and 0.32 eV, respectively, see Supplementary Figure 14b.

Supplementary Note 12. DFT-calculated adsorption energies and OER activity of RuO_2

It is a well-known experimental fact that RuO_2 is more active than IrO_2 for the OER (see ref. 25, for instance). However, Scheffler et al.[26] pointed out that standard DFT has problems in the description of adsorption energetics at RuO_2(110) surfaces. In particular for the OER, Kitchin, Rossmeisl and coworkers,[27,28] have shown that such inaccuracies result in the prediction of large overpotentials for RuO_2, which can be mitigated by the use of DFT+U. In a very recent work devoted to the OER activities of IrO_2 and RuO_2, Briquet et al.[29] showed that the two oxides bind *O with similar strength, but RuO_2 anomalously binds *OH and *OOH more weakly by ~0.6 eV. Briquet et al. did not use DFT+U in their work, but they also found a solution to the problem by means of explicit solvation in combination with van der Waals-type functionals. For simplicity, in this study we used DFT+U with the U value recommended by Kitchin et al. for Ru.[28] Such calculation setup provides similar overpotentials for RuO_2 (0.60 V) and IrO_2 (0.56 V) which, in view of the ± 0.2 V uncertainty of volcano plots, is compatible with the experimental observation that RuO_2 is more active than IrO_2.

Supplementary Note 13. Cyclic voltammograms during the OER

We studied the durability of the samples by measuring several (up to 80) OER cycles. Doping with Na significantly improves the stability of the catalysts. Supplementary Figure 16 shows selected raw currents measured during the first 20 cycles of reaction. $SrRuO_3$ loses more than 85% of its initial activity while Na-doped samples only 15%. Supplementary Figure 17a shows the evolution of the OER activity during 80 cycles, where Na samples still keep around 30% of their activity.

Also, note that the double-layer region of $SrRuO_3$ decreases upon cycling whereas it remains roughly constant for $Sr_{1-x}Na_xRuO_3$. As discussed in the main text, corrosion during OER results in the surface reconstruction of the $SrRuO_3$ and the observation of RuO_x particles deposited at the surface of the catalysts. This surface reconstruction is responsible for the modificaiton of the double-layer region, which, as obseved in Supplementary Figure 16, is significant in $SrRuO_3$, but very limited in the Na-doped perovskite.

Supplementary Note 14. Characterization of Used Samples

We have analyzed Sr and Ru content in the electrolyte before and during the OER reaction with $SrRuO_3$ and $Sr_{0.90}Na_{0.10}RuO_3$. For this experiment, we used a 1 mm thin graphite wafer (geometric surface of 500 mm^2) as working electrode and we deposited 5 mg of electrocatalyst in both faces. We collected electrolyte before the reaction and after 10, 40 and 80 OER cycles. The evolution of Sr and Ru concentrations in the electrolyte during the OER with both catalysts is shown Supplementary Figure 17b. As observed, Sr and Ru dissolve faster in $SrRuO_3$ than in $Sr_{0.90}Na_{0.10}RuO_3$, with cycling, indicating that the Na-doped sample is more stable for the OER in acid electrolyte. This observation is in perfect agreement with the results shown in Supplementary Figure 16, showing that the stability of Na doped samples is enhanced compared to $SrRuO_3$. Supplementary Figure 17b also shows that the dissolution of Sr is higher than that of Ru in both catalysts (especially in $SrRuO_3$), confirming that Sr dissolution leads to the formation of RuO_x nanoparticles at the surface of the perovskites.

Supplementary Figure 18 shows TEM and HRTEM images of $SrRuO_3$ catalyst after 20 OER cycles. As can be seen in the HRTEM image and in its corresponding DDP, a significant loss of crystallinity of the perovskite structure occurs. The particles are not single crystals anymore, but present disoriented nano-domains, few of them still maintaining the initial perovskite structure, but also showing amorphous areas. We believe that the collapse of the perovskite structure due to the Sr dissolution during OER leads to the formation of ~RuO_x nanoparticles. Its formation is directly correlated with a decay of the activity of the catalyst. We have also cycled and performed TEM of the doped samples after 80 cycles of reaction and part of the sample is converted onto RuO_x nanoparticles. The analysis of $Sr_{0.90}Na_{0.10}RuO_3$ recovered after 20 cycles in the OER (Supplementary Figure 19) reveals that the Na doped perovskite is crystalline after OER, in striking contrast with the observation for $SrRuO_3$ (Supplementary Figure 18).

We have measured XRD before and after 20 cycles of OER reaction (Supplementary Figure 20). We observe that the perovskite phase is the only crystalline component in both samples before and after 20 cycles of reaction. We also observe that the amorphous component in the diffractograms increases with cycling indicating that other phases are appearing but they are not crystalline, as observed by TEM and XAS (see below).

Samples measured before and after 20 cycles show clear differences in the Ru K-edge XAS signal (Supplementary Figure 21). The undoped sample after 20 cycles shows a high degradation

of the perovskite structure, which reflects in an increase of XANES pre-edge features and decrease of white line and subsequent peaks (Supplementary Figure 21a, pink line). In addition, Fourier Transform curves show a decrease in the intensity of peaks related to perovskite structure and appearance of features that can be attributed to RuO_2 local formation (Supplementary Figure 21b, pink line). A linear combination fit on the EXAFS signal gives an approximate estimation of the RuO_2 to $SrRuO_3$ ratio of 80:20 (Supplementary Figure 21c). The doped sample after 20 cycles shows that the perovskite structure is almost unchanged, both XANES and EXAFS signature features for the $Sr_{0.90}Na_{0.10}RuO_3$ structure are still present at high distances (Supplementary Figure 21a and b, dark blue line). Performing the same linear combination fit used for the undoped sample, in this case the ratio is inverted, so 80% of the sample is still perovskite and the signal that can be attributed to RuO_2 is limited to less than 20% (Supplementary Figure 21d).

Supplementary Note 15. Assessment of mass transfer effect

It has been reported that using a high catalyst loading, and consequently a thick layer of catalyst, could result in a distortion of the laminar flow and consequently to mass transfer issues in the ORR.[6] We have assessed the effect of catalyst loading on the electrode by conducting several experiments. First, we decreased catalyst loading on our electrode by half; obtaining roughly half currents (40% lower) indicating the lack of mass transfer issues, see Supplementary Figure 22a. Next, we recorded the OER with and without rotation obtaining similar currents, which indicates that the OER is not controlled by diffusion (Supplementary Figure 22b). Finally, we have conducted the OER at different scan rates 1 and 10 mVs^{-1}. As observed in Supplementary Figure 22c, the currents obtained are similar at both scan rates which, according to previous reports, indicates the absence of mass transfer effects during the OER, see [7] and references therein.

Supplementary References

1 Kim, J.-Y. *et al.* 4d Electronic structure analysis of ruthenium in the perovskite oxides by Ru K- and L-edge XAS. *J. Synchrotron Rad.* 8, 722-724 (2001).
2 Suntivich, J., Gasteiger, H. A., Yabuuchi, N. & Shao-Horn, Y. Electrocatalytic Measurement Methodology of Oxide Catalysts Using a Thin-Film Rotating Disk Electrode. *Journal of The Electrochemical Society* 157, B1263 (2010).
3 Trasatti, S. & Petri, O. A. Real surface area measurements in electrochemistry. *Pure & Appl. Chern.* 63, 71 71-734 (1991).
4 Cheng, X. *et al.* Oxygen Evolution Reaction on La1–xSrxCoO3 Perovskites: A Combined Experimental and Theoretical Study of Their Structural, Electronic, and Electrochemical Properties. *Chemistry of Materials* 27, 7662-7672 (2015).
5 McCrory, C. C., Jung, S., Peters, J. C. & Jaramillo, T. F. Benchmarking heterogeneous electrocatalysts for the oxygen evolution reaction. *J Am Chem Soc* 135, 16977-16987 (2013).
6 Paulus, U. A., Schmidt, T. J., Gasteiger, H. A. & Behm, R. J. Oxygen reduction on a high-surface area Pt/Vulcan carbon catalyst: a thin-film rotating ring-disk electrode study *Journal of Electroanalytical Chemistry* 495, 134–145 (2001).
7 Han, X. *et al.* Mass and Charge Transfer Coenhanced Oxygen Evolution Behaviors in CoFe-Layered Double Hydroxide Assembled on Graphene. *Advanced Materials Interfaces* 3, 1500782 (2016).
8 Chang, S. H. *et al.* Functional links between stability and reactivity of strontium ruthenate single crystals during oxygen evolution. *Nat Commun* 5, 4191 (2014).
9 Lee, Y.-L., Kleis, J., Rossmeisl, J. & Morgan, D. Ab initio energetics of LaBO3(001) (B = Mn, Fe, Co, and Ni) for solid oxide fuel cell cathodes. *Physical Review B* 80, 224101 (2009).
10 Mowbray, D. J. *et al.* Trends in Metal Oxide Stability for Nanorods, Nanotubes, and Surfaces. *The Journal of Physical Chemistry C* 115, 2244-2252 (2011).
11 Eichler, A., Hafner, J., Furthmüller, J. & Kresse, G. Structural and electronic properties of rhodium surfaces: an ab initio approach. *Surface Science* 346, 300-321 (1996).
12 Man, I. C. *et al.* Universality in Oxygen Evolution Electrocatalysis on Oxide Surfaces. *ChemCatChem* 3, 1159-1165 (2011).
13 Calle-Vallejo, F., Díaz-Morales, O. A., Kolb, M. J. & Koper, M. T. M. Why Is Bulk Thermochemistry a Good Descriptor for the Electrocatalytic Activity of Transition Metal Oxides? *ACS Catalysis* 5, 869-873 (2015).
14 Pourbaix, M. Atlas of electrochemical equilibria in aqueous solutions. (1974).
15 Calle-Vallejo, F., Martinez, J. I., Garcia-Lastra, J. M., Mogensen, M. & Rossmeisl, J. Trends in stability of perovskite oxides. *Angew Chem Int Ed Engl* 49, 7699-7701, doi:10.1002/anie.201002301 (2010).
16 Nørskov, J. K. *et al.* Origin of the Overpotential for Oxygen Reduction at a Fuel-Cell Cathode. *J. Phys. Chem. B* 108, 17886-17892 (2004).
17 Seitz, L. C. *et al.* A highly active and stable IrOx/SrIrO3 catalyst for the oxygen evolution reaction. *Science* 353, 1011-1014 (2016).
18 Diaz-Morales, O., Ledezma-Yanez, I., Koper, M. T. M. & Calle-Vallejo, F. Guidelines for the Rational Design of Ni-Based Double Hydroxide Electrocatalysts for the Oxygen Evolution Reaction. *ACS Catalysis* 5, 5380-5387 (2015).
19 Vojvodic, A. *et al.* On the behavior of Brønsted-Evans-Polanyi relations for transition metal oxides. *The Journal of Chemical Physics* 134, 244509 (2011).

20 Govindarajan, N., García-Lastra, J. M., Meijer, E. J. & Calle-Vallejo, F. Does the breaking of adsorption-energy scaling relations guarantee enhanced electrocatalysis? *Current Opinion in Electrochemistry*, 1-8 (2018).
21 Quaino, P., Juarez, F., Santos, E. & Schmickler, W. Volcano plots in hydrogen electrocatalysis - uses and abuses. *Beilstein J Nanotechnol* 5, 846-854 (2014).
22 Nørskov, J. K. *et al.* Trends in the Exchange Current for Hydrogen Evolution. *Journal of The Electrochemical Society* 152 (2005).
23 Rossmeisl, J., Qu, Z. W., Zhu, H., Kroes, G. J. & Nørskov, J. K. Electrolysis of water on oxide surfaces. *Journal of Electroanalytical Chemistry* 607, 83-89 (2007).
24 Appleby, A. J. Electrocatalysis and Fuel Cells. *Catalysis Reviews* 4, 221-244 (1971).
25 Stoerzinger, K. A., Qiao, L., Biegalski, M. D. & Shao-Horn, Y. Orientation-Dependent Oxygen Evolution Activities of Rutile IrO2 and RuO2. *Journal of Physical Chemistry Letters* 5, 1636-1641 (2014).
26 Kiejna, A. *et al.* Comparison of the full-potential and frozen-core approximation approaches to density-functional calculations of surfaces. *Physical Review B* 73, 035404 (2006).
27 Xu, Z. & Kitchin, J. R. Tuning oxide activity through modification of the crystal and electronic structure: from strain to potential polymorphs. *Physical Chemistry Chemical Physics* 17, 28943-28949 (2015).
28 Xu, Z., Rossmeisl, J. & Kitchin, J. R. A Linear Response DFT+UStudy of Trends in the Oxygen Evolution Activity of Transition Metal Rutile Dioxides. *J. Phys. Chem. C* 119, 4827-4833 (2015).
29 Briquet, L. G. V., Sarwar, M., Mugo, J., Jones, G. & Calle-Vallejo, F. A New Type of Scaling Relations to Assess the Accuracy of Computational Predictions of Catalytic Activities Applied to the Oxygen Evolution Reaction. *ChemCatChem* 9, 1261-1268 (2017).
30 Calle-Vallejo, F. *et al.* Number of outer electrons as descriptor for adsorption processes on transition metals and their oxides. *Chem. Sci.* 4, 1245 (2013).
31 Grimaud, A. *et al.* Double perovskites as a family of highly active catalysts for oxygen evolution in alkaline solution. *Nat Commun* 4, 2439 (2013).
